# Phylogeography of Poorly Dispersing Net-Winged Beetles: A Role of Drifting India in the Origin of Afrotropical and Oriental Fauna

**DOI:** 10.1371/journal.pone.0067957

**Published:** 2013-06-26

**Authors:** Katerina Sklenarova, Douglas Chesters, Ladislav Bocak

**Affiliations:** Department of Zoology, Faculty of Science, Palacky University, Olomouc, Czech Republic; BiK-F Biodiversity and Climate Research Center, Germany

## Abstract

Ancient dispersal history may be obscured by subsequent dispersal events. Therefore, we intend to investigate the biogeography of metriorrhynchine net-winged beetles, a group characterized by limited dispersal propensity. We used DNA data to construct phylogenies and the BayesTraits and RASP programs to identify putative ancestral areas. Further, we inferred ultrametric trees to estimate the ages of selected nodes. The time frame is inferred from tectonic calibrations and the general mutation rate of the mitochondrial genes. Metriorrhynchini consists of two lineages with Afro/Oriental and Australian distributions. The basal lineages originated in Eastern Gondwana after the split of Australia, India and Madagascar; the Afrotropical and Madagascar Metriorrhynchini separated from the Oriental clades 65 and 62 mya. Several already diversified lineages colonized continental Asia 55–35 mya. A few genera of the Australian clade dispersed to the Oriental region 5–15 mya and reached Eastern India and Southern China. Only *Xylobanus* crossed the Makassar Strait to Sulawesi and does not occur further to the east. The current distribution of Metriorrhynchini is a result of drifting on continental fragments and over-sea dispersal events limited to a few hundreds of kilometers. We conclude that: (1) Afrotropical and Madagascar lineages originated independently from dispersal events during India's drift to the north and the Mozambique Channel completely isolates the respective faunas since then; (2) Oriental fauna is a recently established mixture of the Indian and Australian lineages, with predominance of the older Indian clades; (3) The fauna of islands located north of Australia colonized Sulawesi after collision with the Sundaland margin and the species rich Australian lineages did not reach Western Wallacea or the Philippines. Our results suggest an impact of subtle differences in biological characteristics on biogeographic history of individual lineages, when mostly lowland and flower-visiting lineages were able to disperse across sea channels.

## Introduction

The structure of biotas of the continents that rim the Indian Ocean are the result of the break-up of Gondwana, connectivity between India, Madagascar and Africa, the collision of the Indian subcontinent with Asia, and the formation of the islands of Wallacea [1], [2], [3], [4], [5], [6]. Studies on dispersal and vicariance history often bring conflicting conclusions depending on the biology of the studied groups as recent dispersals and extinctions can easily obscure older patterns [7], [8]. Here, we use poorly dispersing net-winged beetles to study the biotic connectivity among Gondwanan continents and Asia.

The dispersal propensity determines the evolution and distribution of many animal lineages. Some groups are well known as wide dispersers, e.g. diving beetles [9], and are often uninformative in phylogeographic studies addressing ancient zoogeographical patterns. Such studies require widespread and species rich model groups with limited dispersal ability. Further, lineages with an uninterrupted long-term diversification are preferable to avoid confounding impacts of extinctions. The metriorrhynchine beetles are a highly diverse Palaeotropical lineage with ∼1400 species and their origin was hypothesized in the Late Cretaceous [10], [11]. Low dispersal ability of net-winged beetles is due to strong dependence on rain forest habitats, weak short-distance flight, short adult life span, and the presence of geographically limited aposematic patterns [12], [13]. Their diversity and small ranges make Metriorrhynchini a promising model group with a potential to elucidate ancient dispersal histories.

**Figure 1:**
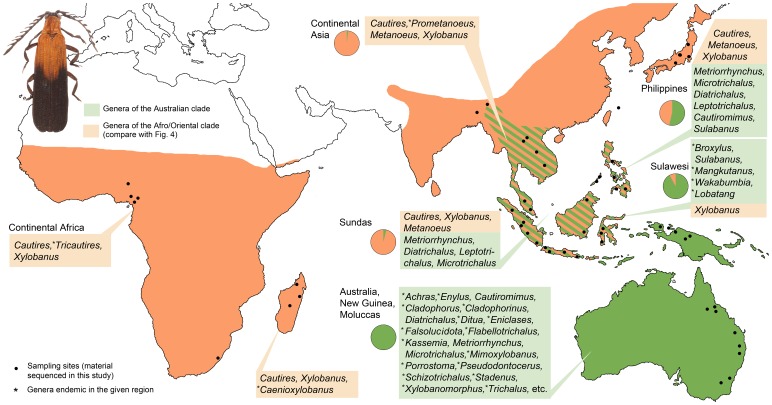
Distribution of Metriorrhynchini. All genera of the Australian clade occurring in the Philippines are also present in Sulawesi.

The morphology-based phylogeny of Metriorrhynchini was presented by Bocak [10] and since then information has been accumulating on the diversity of the lineage [14], [15]. An apparent trait of the lineage is high morphological diversity in the Australian region, namely in New Guinea and humid areas of Australia where 22 endemic genera occur; some of them, e.g. *Porrostoma* and *Cladophorus*, are represented by hundreds of species [10]. In contrast, only three genera *Cautires*, *Xylobanus*, and *Metanoeus* represent almost complete species-level richness of the Oriental and Afrotropical fauna (altogether ∼600 spp., Fig. 1). A few genera occur on both sides of the Wallace line and in all cases the number of species is highly asymmetrical, with the majority of species known from either region (Fig. 1).

The wide distribution of Metriorrhynchini raises the question of how they came to occupy such a range. We expect that drifting on continental fragments beside dispersal played an important role, as their origin is placed well after the breakup of Gondwana [11]. There are several aims of this study: (1) Reconstruct phylogenetic relationships; (2) Identify the areas of origin and present the first worldwide biogeographical analysis of the dispersal routes of Metriorrhynchini in conjunction with plate tectonics [1], [5], [6]; and (3) Investigate the phylogenetic structure of the hyperdiverse Oriental fauna. The impact of ecological characteristics on biogeographic history is discussed.

## Materials and Methods

### Sampling, DNA Extraction, PCR Amplification, and Sequencing

Altogether 226 samples of Metriorrhynchini representing ∼170 species from all regions from their range were sampled (Fig. 1) with Genbank accession numbers listed in Tab. S1. DNA was extracted using the Wizard SV96 Purification System (Promega Inc.). Extraction yield was measured using a NanoDrop-1000 Spectrophotometer. The PCR settings and cycle sequencing conditions used were as reported by Malohlava & Bocak [16]. Five fragments were sequenced: the 18S rRNA (∼1900 base pairs, bp), the D2 region of the 28S rRNA (∼630 bp), *rrnL* mtDNA, *tRNA-Leu* with partial *nad1* (∼780 bp), *cox1*, *tRNA-Leu* and *cox2* mtDNA (1100 bp), and 1180 bp of *nad5* mtDNA with adjacent *tRNA-Phe*, *tRNA-Glu*, and *tRNA-Ser* (multiple gene fragments are referred as *rrnL*, *cox1*, and *nad5* further). The primers used are listed in Tab. S2. The PCR products were purified using PCRu96 Plates (Millipore Inc.) and sequenced by an ABI 3130 automated sequencer using the Big Dye Sequencing Kit 1.1.

### Sequence Handling and Phylogenetic Analyses

Sequences were edited using Sequencher 4.10.1 (Gene Codes Corp.). Protein-coding genes contained few indels and were aligned by ClustalW 1.83 [17]. Length variable loci were separately aligned using four methods: ClustalW 1.83 using penalties 22.5 for gap opening and 0.83 for extension, T-coffee 8.95 [18], Mafft v. 7 [19] and BlastAlign 1.2 [20], all under default parameters, and Muscle 3.6 [21] under the gap opening parameter −600 and gap extension parameter −40. The concatenated supermatrices combined the length variable fragments aligned using various methods and protein coding mtDNA fragments aligned using ClustalW. The alignments were deposited to the Dryad database.

Phylogenies were inferred using Parsimony (MP), Maximum Likelihood (ML) and Bayesian Inference (BI) algorithms. The MP analysis was carried out using TNT 1.1 [22]. For ML trees we used RAxML 7.2.5 [23], with separate parameters applied to the 18 partitions (Tab. S3). Confidence was determined with 100 bootstrap replicates utilizing the rapid bootstrap option under the GTRCAT substitution model as given by the AICc criterion in jModelTest 3.7 [24]. Additionally, the dataset was analyzed using MrBayes 3.2.1 [25]. The MCMC was set with independent parameters for 18 partitions under the general time reversible model with a category of invariant sites and gamma distributed rates (GTR+I+G). Four chains were run for 40.10^6^ generations, with trees sampled every 1,000 generations. The stationary phase was detected using Tracer 1.5 [26], pre-stationary trees were discarded as the burn-in phase and posterior probabilities determined from the remaining trees.

A likelihood ratio test was used to test the molecular clock hypothesis. Under the null hypothesis L_0_, the molecular clock holds, while hypothesis L_1_ imposes no clock constraint. The chi-square value is given by 2logL = 2(logL_0_-logL_1_) where L_0_ and L_1_ are likelihoods of the tree under the given model, and the p-value is calculated for s-2 degrees of freedom where s is number of terminal branches on the tree [27].

### Historical Biogeography Analyses

**Figure 2:**
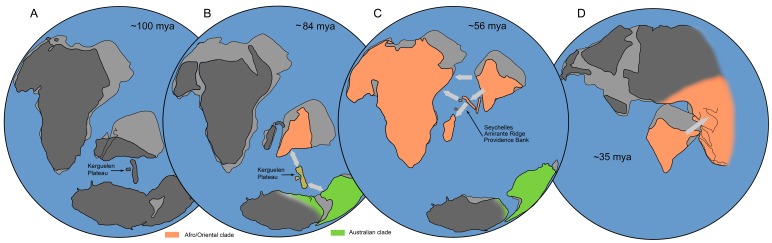
Schematic positions of the Gondwanan continents (A) before the origin of Metriorrhynchini, (B) at the time of the basal split, (C) at the time of dispersal to Africa and Madagascar and (D) at the time of dispersal to continental Asia. Position of continents redrawn from Ali & Aitchison (2008), position of India in Fig. 2D from [47]. Colored areas depict hypothesized ranges of the Afro/Asian and Australian clade; arrows indicate presumed dispersal events.

The absence of the net-winged beetle fossils makes any calibration difficult, and therefore two calibrating points and substitution rate were employed to date splits of interest: (i) The basal split in Metriorrhynchini at ∼78 mya inferred from the divergence of Lycini and Calopterini in the dated phylogeny of Lycidae [11]; (ii) Alternatively, we used the arbitrary point 100 mya, when the latest presence of the Kerguelen Plateau [5] could support connection between India and Australia (Fig. 2A); (iii) Finally, the mean substitution rate of mtDNA was fixed to 0.0115 substitutions/lineage/my, which appeared to be satisfactory in *Metriorrhynchus* [13]. The mean rate is a composite rate of rapidly and slowly evolving genes [28].

We estimated the time to the most recent common ancestor for selected clades using a Bayesian approach implemented in Beast 1.6.1 [29]. All analyses were performed using a GTR+I+G model as given by the AICc criterion in jModelTest 3.7 [24], using a relaxed molecular clock and an uncorrelated lognormal model of rate variation among branches. The data were partitioned (Tab. S3), with each partition allowed independent parameters. In all analyses 12.10^7^ generations were run and trees sampled every 1,000 generations. Convergence was assessed in Tracer 1.5 [26]. The mtDNA dataset was run four times and results combined.

Using the BI trees, we were interested in the reconstruction of ancestral geographical states at key internal nodes. In total, nine geographic regions were defined: Australia, Sulawesi, the Philippines, Palawan, Madagascar, Africa, the Western Malay Archipelago (Sumatra, Borneo, Java and the Malay Peninsula; referred as Sundas further), continental Asia north of the Kra Isthmus, and an external region for the outgroup, as the sister-group of Metriorrhynchini is unknown. The geographic states at each terminal were input into BayesTraits [30] and the likelihood of each alternative geographic state inferred for nodes of interest. Nodes to be reconstructed were defined on the Bayesian consensus topology. Due to the time consuming nature of defining internal nodes for reconstruction, a Perl script was developed to read newick format trees and prepare the appropriate BayesTraits input commands. The script is made freely available online (https://sourceforge.net/projects/bayestraitswrap/). In order to account for phylogenetic uncertainty, ancestral reconstruction was performed for a number of trees sampled during the stationary phase of the Bayesian search. The likelihoods of each state were calculated where a node of the consensus tree was present in the given sampled tree (BayesTraits command: AddNode), then the average likelihoods calculated over each of 30 sampled trees. We used the MP-based statistical dispersal-vicariance analysis implemented in RASP 2.1 [31] for an alternative ancestral state reconstruction. We randomly selected 1000 Bayesian trees inferred from the Muscle alignment after burning the non-stationary phase; the geographical regions were coded as above.

## Results

### DNA Sequencing and Estimation of Phylogeny

DNA sequences were obtained for five fragments (Tab. S2). The rRNA fragments varied in length, with four alignment algorithms producing datasets of 5792–5963 characters. The numbers of characters and their informativeness are given in Tab. S3.

**Figure 3:**
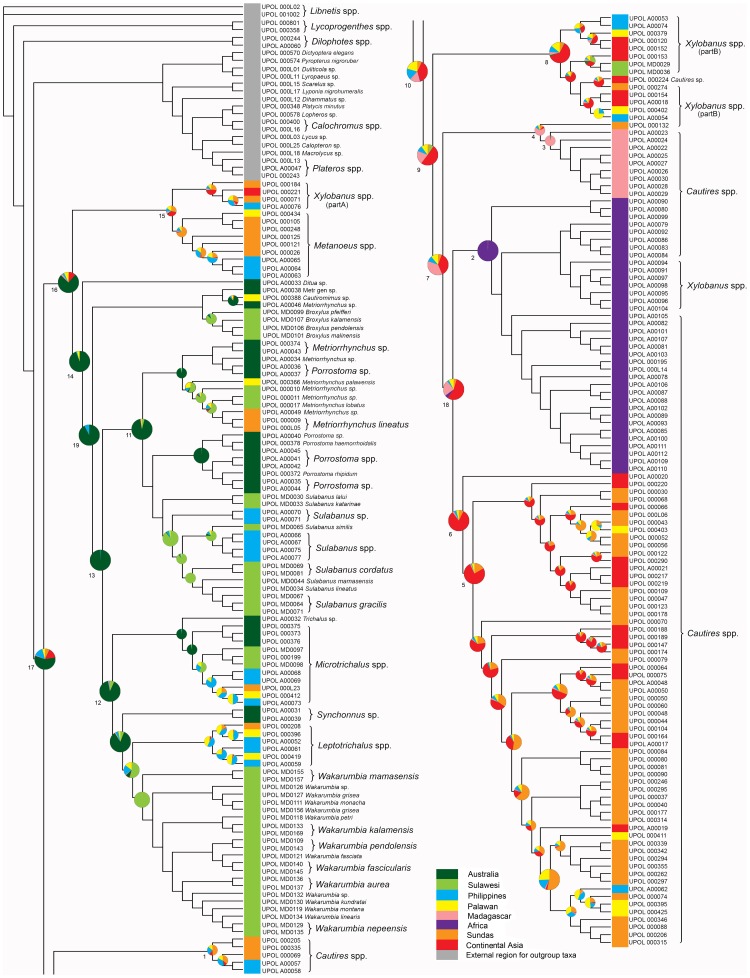
Phylogenetic hypothesis for Metriorrhynchini based on a ML analysis of all available fragments (18S and 28S rRNA, *cox1*, *nad5* and *rrnL* mtDNA). Numbers at the branches designate clades listed in Tab. 3. The charts indicate probabilities of ancestral areas inferred from the BayesTraits analysis.

The phylogenetic reconstruction inferred by MP, ML, and BI resulted in similar topologies for the concatenated datasets inferred under the five alignment algorithms (Figs. 3, S1). The trees had fully resolved basal branches, although the arrangement of some clades varied across analyses (Tab. 1).

**Table 1:** Nodes recovered by analyses of the datasets produced by five alignment procedures using parsimony (MP), maximum likelihood (ML) and Bayesian (BI) algorithms.

Alignment	BlastAlign			Clustal			Muscle			Tcoffee			Mafft		
Algorithm	MP	ML	BY	MP	ML	BY	MP	ML	BY	MP	ML	BY	MP	ML	BY
Metriorrhynchini	M	M	M	M	M	M	M	M	M	M	M	M	M	M	M
*Metanoeus(Xylobanus,Cautires)*	P	M	M	P	M	M	P	P	P	P	M	M	P	P	M
(*Metanoeus*,*Xylobanus* partA)	M	M	M	M	M	M	M	M	M	M	M	M	M	M	M
(*Xylobanus* partB, *Cautires*)	M	M	M	M	M	M	M	M	M	M	M	M	M	M	M
(Australian Metriorrhynchini)	M	–	M	M	–	M	M	M	M	M	M	M	M	M	M
(*Sulab.*(Austr. Metriorh.part)	–	M	–	M	M	–	–	M	M	M	M	M	M	–	–
(*Synch*., *Leptotr., Wakarumbia)*	M	M	M	–	M	M	–	M	M	–	M	M	M	M	M
(*Porrostoma*, *Metriorrhynchus*)	–	M	M	M	M	–	–	M	M	–	M	M	P	M	M
(*Trichalus*, *Microtrichalus*)	M	M	M	M	M	M	–	–	M	–	M	M	M	M	M

**Figure 4:**
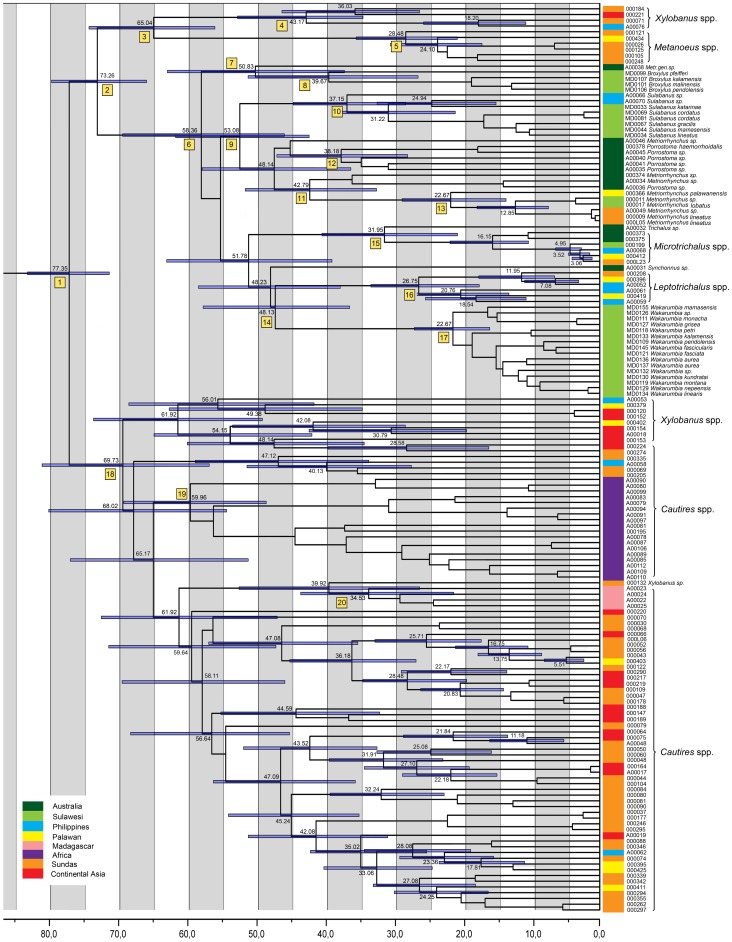
Timing of the Metriorrhynchini radiation. Estimated mean ages of nodes are based on Bayesian analysis of all fragments under the relaxed molecular clock model and the root calibrated at 77.7 mya. The bars depict 95% confidence intervals; the numbers at the branches designate clades listed in Tab. 2.

All trees indicate that Metriorrhynchini represent a monophyletic clade. Three clades were consistently found as basal splits: *Xylobanus*(A)+*Metanoeus*, the Australian clade (*Sulabanus*, *Wakarumbia*, *Porrostoma*, *Metriorrhynchus*, *Microtrichalus*, etc.) and the Afro/Oriental clade (*Xylobanus*(B)+*Cautires*; both genera inferred as a paraphyletic assemblage). The first clade was found as a sister group of either the Australian or Afro/Oriental clade (Tab. 1, Figs. 3–4, Figs. S1–S2). The unconstrained Beast analysis suggested the first topology (Fig. 4). The clade designations refer to their ancestral ranges and are discussed under their respective names further.

### Divergence Times and Ancestral Areas

The presence of a molecular clock was tested for using a likelihood ratio test with the ClustalW alignment. The likelihood under a model with no clock constraint was −212138, and −213384 with a clock constraint, giving a likelihood ratio of 1246 (2*(213384–212138)), a significant rejection of the molecular clock at d.f. of 249 and significance cutoff of 0.05.

Therefore, speciation events were dated using a relaxed molecular clock as implemented in Beast 1.6.1. Due to uncertainties in molecular dating, we used two tectonic-based calibrations and a general molecular rate as described in the Methods. The normalized tree calibrated by the age of the Metriorrhynchini (∼78 mya) sets a time of dispersal to Africa at ∼65 mya, and to Madagascar ∼62 mya (Fig. 4, Tab. 2). The dispersal events across the Wallace line and to the Philippines are inferred in the congruence with the tectonic history (Fig. 4). The alternative calibrations proposed deeper (when calibrated by the latest presence of the subaerial Kerguelen Plateau) or slightly shallower (calibrated by mutation rate) dating. The complete results are given in Tab. 2.

**Table 2:** Estimation of the age of selected nodes inferred from the Bayesian.

Clade number in Fig. 4		Root fixed at 77.7. ±3.02 my		Rate 0.0015% my^−1^		Root fixed at 100 mya	
	Taxon/node	mean (my)	95% HPD	mean (my)	95% HPD	mean (my)	95% HPD
1	Metriorrhychini (root)	77.35*	71.44–83.22	73.57	63.88–82.81	99.74*	93.86–105.73
2	Austr.(*Xylob*.+*Metan*.)	73.26	66.55–79.97	68.03	61.15–75.32	94.37	86.23–102.93
3	*Xylobanus*(2)+*Metanoeus*	65.04	55.54–75.76	61.92	50.70–72.50	83.34	74.17–92.46
4	*Xylobanus* (clade 1)	43.17	34.70–51.56	41.28	31.06–50.30	55.49	45.12–65.78
5	*Metanoeus*	28.48	21.07–35.71	27.36	20.84–34.14	37.30	28.89–46.57
6	Metriorrhynchini (austr.)	58.36	47.57–67.78	55.90	48.28–62.25	77.37	67.92–86.36
7	*Cautiromimus*+*Broxylus*	50.83	37.50–67.09	49.03	37.65–61.20	65.97	50.83–84.45
8	*Broxylus*	39.67	28.82–51.12	38.73	28.82–47.80	51.95	39.23–68.81
9	*Porrost.*+*Sulab.*+*Metr*.	53.08	42.69–63.70	50.52	43.80–57.34	68.98	59.16–79.19
10	*Sulabanus*	37.15	28.22–45.77	35.78	29.01–42.56	48.25	38.79–58.12
11	*Metriorrhynchus*	42.79	32.74–52.89	40.71	33.25–48.58	55.42	43.87–66.51
12	*Porrostoma*	38.18	28.56–47.46	36.34	28.80–44.05	49.82	39.03–60.14
13	*Metriorrhychus* (Sulaw.)	22.67	15.19–30.46	21.58	15.56–28.09	29.77	21.18–39.12
14	*Leptotrich*.+*Wakarumbia*	48.13	38.41–58.59	45.90	38.61–53.57	62.97	52.60–73.84
15	*Trichalus*	31.95	22.23–41.49	31.11	22.51–40.15	42.12	30.23–54.58
16	*Leptotrichalus*	26.75	19.44–34.38	25.45	19.22–31.75	35.12	26.04–43.78
17	*Wakarumbia*	22.67	17.36–28.38	21.34	17.33–25.88	29.02	23.03–35.20
18	*Xylobanus*+*Cautires*	69.73	59.56–79.40	66.43	60.42–72.73	90.99	83.14–98.60
19	*Cautires* (Afrotropical)	59.96	49.58–69.71	57.11	50.59–63.79	78.06	68.75–87.17
20	*Cautires* (Madagascar)	34.53	24.02–44.99	32.46	24.04–41.45	45.01	33.06–58.23

* Asterisk designates the nodes used for calibration.

The program BayesTraits was used to reconstruct the ancestral area states of key nodes in the Metriorrhynchini phylogeny. The analyses gave strong support for the basal split of the Australian and Afro/Oriental lineages (Fig. 3, Tab. 3, S4). High likelihoods were assigned to separate dispersals to Africa and Madagascar. A limited number of lineages crossed the Wallace's line from Sulawesi to Borneo or the Philippines, or the Huxley's line from Asia to the Philippines. The Makassar Strait proved to be an effective barrier to dispersal, as only *Metriorrhynchus* and *Microtrichalus* crossed the line in the westward direction, and *Xylobanus* in the eastward direction. Similar results were inferred from the statistical dispersal-vicariance analysis (Fig. S2).

**Table 3:** Reconstructed probabilities of nine geographic areas at each of selected nodes of the Bayesian phylogeny.

Node#	Node code	Defined range:									
		Sundas	Sulawesi	Continental Asia	outside	Africa	Australia	Madagascar	Philippines	Palawan	node # in Tab.S4
1	Cautires1	0.48098	0.00626	0.11454	0	0.00006	0.00757	0.03428	0.21455	0.14177	20
2	Cameroon	0	0.00387	0	0	0.98903	0	0.00481	0.00011	0.00209	55
3	Madagascar1	0.00022	0.00381	0.00284	0	0	0.02506	0.96792	0	0	63
4	Madagascar 2	0.08706	0.02045	0.06322	0	0.00219	0.00962	0.66407	0.09364	0.05975	64
5	Cautires2	0.16606	0.00004	0.76362	0	0	0.00006	0.01782	0.01323	0.03917	124
6	Cautires3	0.07343	0.00026	0.82507	0.00001	0	0.00030	0.02258	0.03902	0.03932	125
7	CautiresMadagasc.	0.05744	0.00032	0.37550	0	0.00303	0.00024	0.36568	0.08801	0.10978	127
8	XylobCautires1	0.05814	0.01505	0.61849	0	0	0.01863	0.04806	0.09906	0.14257	140
9	XylobCautires2	0.02893	0.06566	0.50941	0	0.00004	0.00053	0.20388	0.08926	0.10228	141
10	Cautires4	0.07070	0.00157	0.36993	0	0.01400	0.00132	0.15853	0.17732	0.20662	142
11	MetriorrhSulabanus	0	0.03997	0.00012	0	0	0.93317	0.01446	0.00139	0.01085	184
12	TrichalusWakarumb	0.00008	0.06730	0.00025	0.00001	0.00001	0.90288	0.01954	0.00823	0.00170	225
13	TrichWakSul	0.00053	0.00029	0	0	0.00013	0.99138	0.00074	0.00629	0.00064	226
14	TrichMetr	0.00004	0.00004	0.00002	0.00002	0.00017	0.94138	0.00008	0.01453	0.04373	227
15	MetanoeusXylobanus	0.25720	0.03002	0.33029	0.00026	0.00204	0.03302	0.07744	0.11255	0.15719	239
16	MetanoeusMetr-ini	0.00615	0.00216	0.10669	0	0	0.75762	0.02838	0.03024	0.06877	240
17	Metriorhynchini	0.05665	0.01317	0.15450	0.00007	0.00016	0.57861	0.01155	0.14325	0.04203	241
18	Cautires Cameroon	0.04622	0.00002	0.54908	0	0.05274	0.00086	0.24338	0.03006	0.07766	425
19	Trichalus Broxylus	0	0.00023	0	0	0	0.92463	0.00094	0.07371	0.00039	426

Selected nodes are designated in Fig. 3. For further clades see Tab. S4.

## Discussion

### Phylogeny

Here, we propose the first molecular phylogeny of Metriorrhynchini. We confirm their monophyly and basal split into the Afro/Oriental and Australian clade (Figs. 1, 3–4). In contrast to the morphology based study [10], molecular data better resolve the phylogenetic structure of the major clades, with just the position of *Metanoeus* remaining unresolved. Both inferred topologies obtained low support for the position of this clade (Fig. S1). *Xylobanus* and *Cautires* form a monophylum with high support, but both genera were inferred as reciprocically paraphyletic due to multiple origins of the strengthening elytral costae used for the definition of these genera.

### Dispersal Propensity

A critical point for further discussion is dispersal propensity of Metriorrhynchini. The earlier studies shoved their limited ability to cross open sea. Bocak & Yagi [13] identified a single dispersal event across the Makassar Strait at the end of mid-Miocene. The strait is now ∼115 km wide in the narrowest point, but was less than 100 km wide repeatedly during glacial low stands [32]. Surprisingly, a few metriorrhynchines crossed this strait (Fig. 3) [15]. On the other hand, the ∼460 km wide Mozambique Channel was never crossed by any net-winged beetle and the only Lycidae shared between Africa and Madagascar are *Cautires* and *Xylobanus*, which are hypothesized a result of independent dispersal events further (Fig. 3) [33]. This finding is in contrast with frequent dispersal events across the Mozambique Channel in other animal lineages [4]. The low dispersal propensity is additionally supported by very species high turn over between close islands [34] or mountain systems [35], [36].

The present differences between faunas of neighboring landmasses enable an estimation of the open-sea distances, which the metriorrhynchines can cross. Therefore, we conservatively accept in further discussion dispersal events across sea channels narrower than 500 kilometers when the geographical proximity lasts over a long time span. Long-distance dispersals across between continents are not considered.

### Biogeography and Dating

The analyses indicate that (i) Metriorrhynchini are of eastern Gondwanan origin and the geographical structure of its two principal lineages, the Afro/Oriental and Australian clades, has been preserved until present (Figs. 1, 3); (ii) Rafting on continental fragments played an important role, with dispersal limited to crossing sea channels a few hundreds kilometers in width; and that (iii) The direction of dispersal was most likely from drifting India to Africa, Madagascar, and continental Asia, and from Australia to Asia (Figs. 2, 4). Due to low dispersal propensity, the Metriorrhynchini missed dispersal opportunities commonly exploited by other animals. They are absent from New Zealand in contrast with diversity in Australia [10]. Despite presence in the Far East Metriorrhynchini did not disperse to North America via the Transberingian route [37]. Similarly, the connection between Australia and South America effective until the late Eocene [38] was not traversed by Metriorrhynchini. Concerning the very high diversity of Metriorrhynchini in all parts of their range we suggest that these patterns are not the result of extinctions, but demonstrate deep dispersal history.

Three approaches were employed to set the sequence of splits inferred from tree topology into a time frame. We used two tectonic events as calibration points: (i) The age of Metriorrhynchini fixed at 78 mya [11] resulted in the dates in Fig. 4, and the inferred time frame is consistent with tectonics of the East Gondwanan region. (ii) The alternative calibration based on the latest presence of the Kerguelen Plateau at 100 mya suggested an unrealistically early dispersal to the Oriental region and Wallacea (Tab. 2) [5]. (iii) The fixed mean substitution rate provided the shallowest estimations amongst the calibrations used, but not substantially different from the preferred dating (Tab. 2). The 'universal' mean clock is prone to error [28] and was applied here to provide another age limit for estimations. Although the inferred dating based on Lycidae phylogeny is congruent with the known sequence of tectonic events resulting in dispersal opportunities, the exact dating remains open for further investigation and we rely herein only on the information inferred from topology, i.e. sequence of events, combined with maximum time limits.

### The Early Evolution of Metriorrhynchini: the Split of the Australian and Afro/Asian Clades

Metriorrhynchini is a Palaeotropical clade, which began diversification in Eastern Gondwana (Fig. 2A). The absence of Metriorrhynchini in the Neotropics and the delayed presence in Africa and Madagascar as well as the age inferred from the mutation rate refutes Gondwanan vicariance. Therefore, ancient over-sea dispersal after Gondwanan fragmentation is considered. The basal split between the *Metanoeus*, Australian and Afrotropical/Oriental clade is set in time when Indian was much closer to Australia than the other parts of the present Asia (75–100 mya, Fig. 2A–B). Considering the limited dispersal ability, the over-sea dispersal between Australia and India starting its drift to the north (definitively <1000 km) [39] is preferred to the dispersal event between Australia and Asia (>2500 km, Figs. 2A–B). Deeper dating within confidence intervals or the presence of the remnants of the subaerial Kerguelen Plateau allow assumption of the dispersal at the distance <500 km. The phylogenetic reconstructions suggest origin of Metriorrhynchini either in Australia or India (Figs. 3–4, S1–S2) taken the ambiguity of the phylogenetic inference in consideration. The Australian origin is supported by the higher morphological diversity of the Australian clade (Fig. S1). The second hypothesis is more parsimonious assuming the topology in Fig. S2. The direction of dispersal will have to be based on a more extensive data set.

### Late Cretaceous Connectivity between India, Madagascar and Africa

Assuming presence of Metriorrhynchini on the drifting India (Fig. 2), we infer independent Lower Tertiary migrations to Africa (∼65 mya) and Madagascar (∼62 mya) at the early stage of the differentiation of the Afro/Oriental clade. The topologies and dating of the splits between the Indian and Afrotropical/Madagascar clades refute the vicariance hypothesis, as these Gondwanan fragments lost connectivity 100 mya [4], [40], well before the origin of the African and Malagasy clades (Figs. 2, 4). The connectivity of these landmasses between the Maastrichtian and Lower Eocene remains contentious, and an island chain connection [5] or a modified position of India [3], were proposed as alternatives to India's isolation during rifting to the north [2]. The present results support biotic connectivity between India and Africa in this period, across sea channels whose effective width might be lowered by the presence of the islands of the Amirante Ridge (Fig. 2C) [5]. The sampling of Afrotropical metriorrhynchines is limited in the present study and we cannot exclude that further dispersal events from India to Africa may be identified with more comprehensive sampling. Colonization via the migration corridor connecting Asia and Africa through the Arabian Peninsula in the Early Miocene (17–20 mya), which led to faunal and floristic exchanges in other groups [41], [42], did not get any support from our data.

Yoder & Novak [4] demonstrated that most of the Malagasy biota is a result of dispersal events originating from the African coast. Metriorrhynchini, although rejected as an old Gondwanan element in Madagascar, suggest a role of India during its rifting to the north as an ancient source of immigrants. The ecological characteristics prevented lycids from the gradual build up of Madagascar biota across the >400 km wide Mozambique Channel and they remained isolated in Madagascar since the colonization of the island.

### Origin and Phylogenetic Structure of the Oriental Fauna

**Figure 5:**
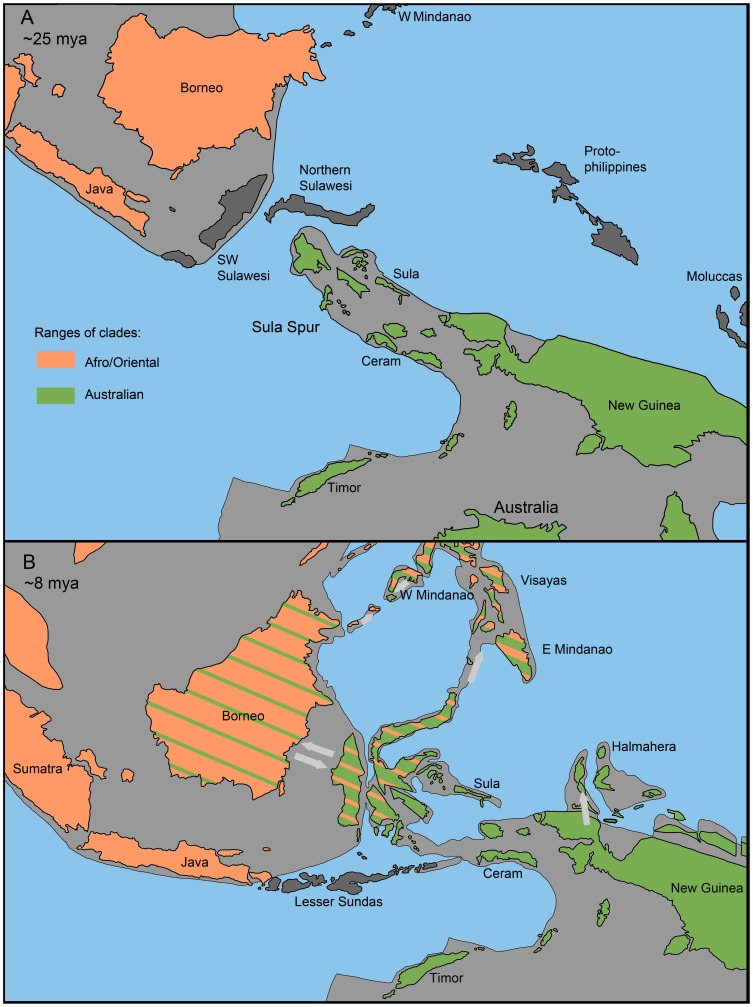
Hypothesized ranges of Metriorrhynchini (A) before the contact of the Australian and Asian continental plates; (B) at time of the dispersal between Sulawesi, Borneo and the Philippines.

Our data imply that Southeast Asian Metriorrhynchini are a phylogenetically heterogeneous assemblage and are formed of two groups: (i) *Cautires*, *Xylobanus*, and *Metanoeus*, which colonized the Oriental Region in Eocene (Fig. 2D, Tab. 2) and (ii), the lineages of the Australian clade (Fig. 5), as recent colonists.

The position of deeply nested lineages which gave an origin to the Madagascar and African fauna supports the India ferry hypothesis as an explanation of the origin of the *Cautires*/*Xylobanus*/*Metanoeus* group in the Oriental region, and refutes later, transoceanic dispersal known in some other animals (the Ninety Ridge hypothesis) [42], [43]. This pattern is congruent with the role of India in the plant dispersal [44]. The clade was well diversified at the moment of dispersal to Asia and we identified 11 (at 55 mya) to 31 (35 mya) lineages in this clade at the time of the collision between India and Asia (Figs. 2D, 4) [5], [45]. The mid Eocene ever-wet rain forest corridor [46] might enable rapid dispersal to east and subsequent diversification (estimated ∼800 species at present). Although Indian plants readily colonized Sulawesi [47], Oriental Metriorrhynchini dispersed there much later and in low numbers (Figs. 3–4), probably during episodes of lower sea levels, which enabled dispersal in the opposite direction [13].

### The Role of Wallacea in Dispersal of Australian Fauna to the North

Wallacea in the present form is a relatively young configuration of islands and the Australian and Oriental lineages could use this dispersal route since the Australian plate approached Asia 15–20 mya (Fig. 5, Tab. 2) [6]. All dispersal events in the region require the assumption of the over-sea dispersal and the distances to cross might have been a few hundreds kilometers as various islands formed and were eroded in the region [1], [6]. Due to rareness of dispersal across such barriers, we still observe substantial differences between the biotas of Wallacea, New Guinea and Northern Australia (Fig. 5). Although effectively used by other organisms [42], these islands provided limited dispersal opportunities for Metriorrhynchini.

Several factors may affect the structure of Metriorrhynchini fauna in Wallacea. The islands at the northern margin of New Guinea moved westward in the last 20 my (Fig. 5); [6], [48] and this might support the dispersal of Australian Metriorrhynchini to west and limit dispersal in the opposite direction. Another important factor are ecological differences: taxa adapted to seasonally semidry habitats and capable of flight outside the rain forest canopy (e.g. flower-visiting *Microtrichalus* and *Metriorrhynchus*) are more widespread. In contrary, the taxa preferring the shaded, moist, under-canopy situations, e.g. *Wakarumbia*, *Sulabanus*, did not disperse across the Makassar Strait. The net-winged beetles exhibit a very conservative life history and even these minor differences in biology have a profound impact on the dispersal success.

Within Wallacea, only the Sulawesi fauna contains representatives of both basal clades (Fig. 1), but the apparent Australian bias in the Sulawesi biota is in contrast with the narrowness of the Makassar Strait (∼100 km depending on the sea levels) [31]. Besides isolation by distance it can be supported by the pronounced seasonality of Sulawesi climate. Although almost completely of Australian origin, the Sulawesi fauna consists mainly of genera, which do not occur in Australia and New Guinea, and are either Wallacea endemics (*Wakarumbia*, *Broxylus*, *Mangkutanus*, and *Lobatang*) or occur additionally in the Philippines (*Sulabanus*) (Fig. 1). The origins of these endemic lineages were dated to 23–40 mya (Fig. 5) and we suppose that they might have a long diversification history in the fragmented Sula Spur [6]. We found that several species rich Australian lineages did not reach Sulawesi and the Philippines (e.g. *Porrostoma*, *Cladophorus*), reached only Sulawesi and the Philippines (*Cautiromimus*) and a few colonized additionally continental Asia (*Metriorrhynchus*, *Microtrichalus*). When present in Sulawesi, these genera are species poor [14], [15] and we suppose their delayed colonization of Wallacea.

### Conclusions

Biological characteristics of the metriorrhynchines have a substantial impact on their evolution. Low dispersal propensity limits Metriorrhynchini to small ranges and even a few hundreds kilometers wide sea channels can limit dispersal between neighboring landmasses. The long-distance dispersal events (>500 km) are absent and repeated expansion of ranges over sea channels were seldom identified. Such immobile organisms provide a distribution pattern that has been historically preserved, and gives a different view on the dispersal history to that of highly mobile animals.

The Indian subcontinent played a central role in the dispersal history of Metriorrhynchini and served as a Noah's Ark, bringing South Gondwanan fauna to Africa (65 mya), Madagascar (62 mya) and Asia (35–55 mya). Australian fauna evolved in complete isolation since the split of the basal lineages until ∼20 mya, when several Australian genera dispersed to Asia (Figs. 2–3, 5).

The Metriorrhynchini are represented by ∼800 species in humid SE Asia and similarly ∼1000 species in New Guinea whose mountain forests have recent origin [44]. Metriorrhynchini are a lineage capable of generating high species level diversity in a very limited space and short time. The Oriental Metriorrhynchini depend on the ever-wet tropical rain forests, and despite geographic proximity only rarely invaded Sulawesi and did not disperse further eastward. In contrast, the Australian flower-visiting or semi-dry condition adapted genera considerably expanded their ranges after crossing sea barriers, and occupy much larger ranges in the Oriental region. These genera occur mostly in dried habitats, including man-disturbed ecosystems.

## Supporting Information

Figure S1 (PDF)

Figure S2 (PDF)

Table S1 (PDF)

Table S2 (PDF)

Table S3 (PDF)

Table S4 (PDF)
