# Supplementary material for: Phylogeography of Poorly Dispersing Net-Winged Beetles: A Role of Drifting India in the Origin of Afrotropical and Oriental Fauna
Source: PLoS One. 2013 Jun 26;8(6):e67957. doi: 10.1371/journal.pone.0067957 (PMC3694047; doi:10.1371/journal.pone.0067957)

Supplementary Figure S1. Phylogenetic hypothesis on Metriorrhynchini inferred from BlastAlign and ML analysis

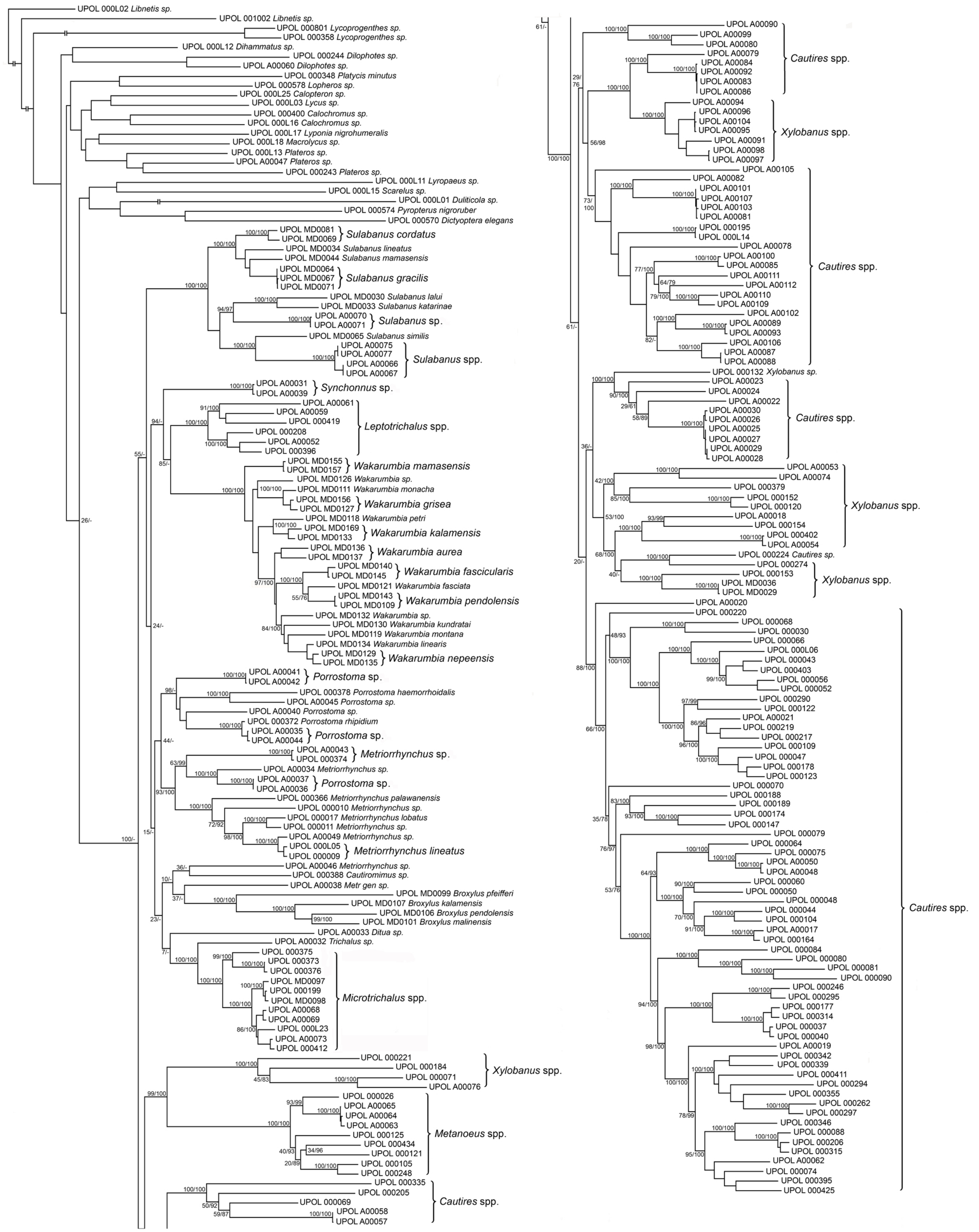

Supplement: Figure S1 — (PDF) [file pone.0067957.s001.pdf]
