# Supplementary material for: Phylogeography of Poorly Dispersing Net-Winged Beetles: A Role of Drifting India in the Origin of Afrotropical and Oriental Fauna
Source: PLoS One. 2013 Jun 26;8(6):e67957. doi: 10.1371/journal.pone.0067957 (PMC3694047; doi:10.1371/journal.pone.0067957)

Supplementary Figure S2. Optimal distributions at each node inferred from the RASP analysis on the set of 1000 Bayesian trees.

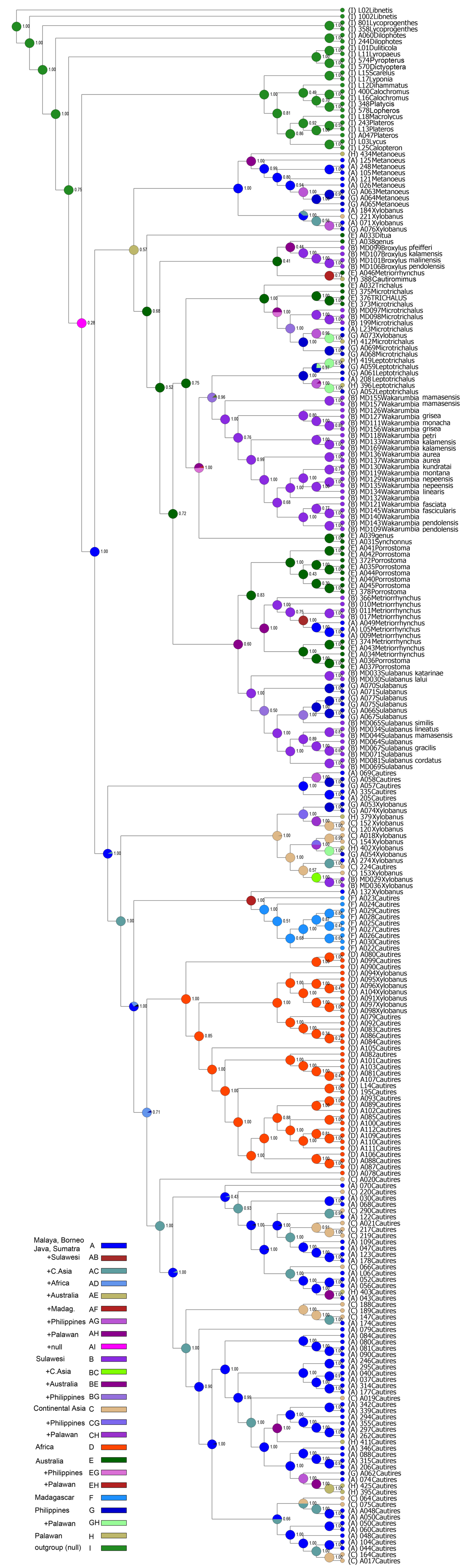

Supplement: Figure S2 — (PDF) [file pone.0067957.s002.pdf]
