# Supplementary material for: Phylogeography of Poorly Dispersing Net-Winged Beetles: A Role of Drifting India in the Origin of Afrotropical and Oriental Fauna
Source: PLoS One. 2013 Jun 26;8(6):e67957. doi: 10.1371/journal.pone.0067957 (PMC3694047; doi:10.1371/journal.pone.0067957)
Supplement: Table S1 — (PDF) [file pone.0067957.s003.pdf]

Table S1. Taxa included in the analysis with collecting information, voucher, and GenBank accession numbers.

Species	Voucher number	Geographic origin	18S	28S	16S	cox1	nad5
Outgroup							
Libnetis sp.	UPOL 001002	Indonesia	DQ181104	DQ181178	DQ181030	DQ181252	DQ181406
Dilophotes sp.	UPOL 000244	Sabah	DQ181066	DQ181140	DQ180992	DQ181214	DQ181368
Plateros sp.	UPOL 000243	Sabah	DQ181065	DQ181139	DQ180991	DQ181213	DQ181367
Platycis minutus	UPOL 000348	Czech Republic	DQ181069	DQ181143	DQ180995	DQ181217	DQ181371
Lycoprogenthes sp.	UPOL 000358	Java	DQ181070	DQ181144	DQ180996	DQ181218	DQ181372
Calochromus sp.	UPOL 000400	Palawan	-	-	-	KC538321	KC538512
Duliticola sp.	UPOL 000L01	Sabah	DQ181037	DQ181111	DQ180963	DQ181185	DQ181339
Libnetis sp.	UPOL 000L02	Sabah	DQ181038	DQ181112	DQ180964	DQ181186	DQ181340
Lycus sp.	UPOL 000L03	South Africa	DQ181039	DQ181113	DQ180965	DQ181187	DQ181341
Calopteron sp.	UPOL 000L25	Ecuador	DQ181053	DQ181127	DQ180979	DQ181201	DQ181355
Lyropaeus sp.	UPOL 000L11	Sabah	DQ181042	DQ181116	DQ180968	DQ181190	DQ181344
Dihammatus sp.	UPOL 000L12	Sabah	DQ181043	DQ181117	DQ180969	DQ181191	DQ181345
Plateros sp.	UPOL 000L13	Sabah	DQ181044	DQ181118	DQ180970	DQ181192	DQ181346
Scarelus sp.	UPOL 000L15	Sabah	DQ181046	DQ181120	KC538782	DQ181194	DQ181348
Calochromus sp.	UPOL 000L16	China	DQ181047	DQ181121	DQ180973	DQ181195	DQ181349
Lyponia nigrohumeralis	UPOL 000L17	China	DQ181048	DQ181122	DQ180974	DQ181196	DQ181350
Macrolycus bocakorum	UPOL 000L18	China	DQ181049	DQ181123	DQ180975	DQ181197	DQ181351
Dictyoptera elegans	UPOL 000570	Japan	DQ181073	DQ181147	DQ180999	DQ181221	DQ181375
Pyropterus nigroruber	UPOL 000574	Japan	DQ181077	DQ181151	DQ181003	DQ181225	DQ181379
Lopheros sp.	UPOL 000578	Japan	DQ181081	DQ181155	DQ181007	DQ181229	DQ181383
Lycoprogenthes sp.	UPOL 000801	Sumatra	DQ181095	DQ181169	DQ181021	DQ181243	DQ181397
Plateros sp.	UPOL A00047	Malaysia	KC538062	KC537854	-	KC538353	KC538544
Dilophotes sp.	UPOL A00060	Philippines	KC538072	KC537863	KC538740	KC538359	KC538552
Ingroup							
Metriorrhynchus lineatus	UPOL 000009	Sumatra	KC538123	KC537913	KC538628	DQ904297	DQ904259
Metriorrhynchus sp.	UPOL 000010	Sulawesi	KC538124	KC537914	-	DQ144659	DQ144685
Metriorrhynchus sp.	UPOL 000011	Sulawesi	KC538125	KC537915	KC538629	DQ144660	DQ144686
Metriorrhynchus lobatus	UPOL 000017	Sulawesi	KC538126	KC537916	KC538630	DQ144662	DQ144688
Metanoeus sp.	UPOL 000026	Borneo	KC538127	KC537917	KC538631	KC538244	KC538436
Cautires sp.	UPOL 000030	Borneo	KC538128	KC537918	KC538632	KC538245	KC538437
Cautires sp.	UPOL 000037	Borneo	KC538129	KC537919	KC538633	KC538246	KC538438
Cautires sp.	UPOL 000040	Borneo	KC538130	KC537920	KC538634	KC538247	KC538439
Cautires sp.	UPOL 000043	Borneo	KC538131	KC537921	KC538635	KC538248	KC538440
Cautires sp.	UPOL 000044	Borneo	KC538132	KC537922	KC538636	KC538249	KC538441
Cautires sp.	UPOL 000047	Sumatra	KC538133	KC537923	KC538637	KC538250	KC538442
Cautires sp.	UPOL 000048	Sumatra	KC538134	KC537924	KC538638	KC538251	KC538443
Cautires sp.	UPOL 000050	Sumatra	KC538135	KC537925	KC538639	KC538252	KC538444
Cautires sp.	UPOL 000052	Sumatra	KC538136	KC537926	KC538640	KC538253	KC538445
Cautires sp.	UPOL 000056	Sumatra	KC538137	KC537927	KC538641	KC538254	KC538446
Cautires sp.	UPOL 000060	Sumatra	KC538138	KC537928	KC538642	KC538255	KC538447
Cautires sp.	UPOL 000064	Laos	KC538139	KC537929	KC538643	KC538256	KC538448
Cautires sp.	UPOL 000066	Laos	KC538140	KC537930	KC538644	KC538257	KC538449
Cautires sp.	UPOL 000068	Borneo	KC538141	KC537931	KC538645	KC538258	KC538450
Cautires sp.	UPOL 000069	Borneo	KC538142	KC537932	KC538646	KC538259	KC538451
Cautires sp.	UPOL 000070	Malaysia	KC538143	KC537933	KC538647	KC538260	KC538452
Xylobanus sp.	UPOL 000071	Borneo	KC538144	KC537934	KC538648	KC538261	KC538453
Cautires sp.	UPOL 000074	Borneo	KC538145	KC537935	KC538649	KC538262	KC538454
Cautires sp.	UPOL 000075	Laos	KC538146	KC537936	-	KC538263	KC538455
Cautires sp.	UPOL 000079	Borneo	KC538147	KC537937	KC538650	KC538264	KC538456
Cautires sp.	UPOL 000080	Borneo	KC538148	KC537938	KC538651	KC538265	KC538457

Cautires sp.	UPOL 000081	Borneo	KC538149	KC537939	KC538652	KC538266	KC538458
Cautires sp.	UPOL 000084	Borneo	KC538150	KC537940	KC538653	KC538267	KC538459
Cautires sp.	UPOL 000088	Malaysia	KC538151	KC537941	KC538654	KC538268	KC538460
Cautires sp.	UPOL 000090	Borneo	KC538152	KC537942	KC538655	KC538269	KC538461
Cautires sp.	UPOL 000104	Borneo	KC538153	KC537943	KC538656	KC538270	KC538462
Metanoeus sp.	UPOL 000105	Borneo	KC538154	KC537944	KC538657	KC538271	KC538463
Cautires sp.	UPOL 000109	Borneo	-	KC537945	KC538658	KC538272	KC538464
Xylobanus sp-	UPOL 000120	Laos	KC538155	KC537946	KC538659	KC538273	KC538465
Metanoeus sp.	UPOL 000121	Sumatra	KC538156	KC537947	KC538660	KC538274	KC538466
Cautires sp.	UPOL 000122	Borneo	KC538157	KC537948	KC538661	KC538275	KC538467
Cautires sp.	UPOL 000123	Java	KC538158	KC537949	-	KC538276	-
Metanoeus sp.	UPOL 000125	Sumatra	KC538159	KC537950	KC538662	KC538277	KC538468
Xylobanus sp.	UPOL 000132	Sumatra	KC538160	KC537951	KC538663	HQ456987	HQ457009
Cautires sp.	UPOL 000147	India	KC538161	KC537952	KC538664	KC538278	KC538470
Xylobanus sp.	UPOL 000152	Laos	KC538162	KC537953	KC538665	KC538279	KC538471
Xylobanus sp.	UPOL 000153	Laos	KC538163	KC537954	KC538666	KC538280	KC538472
Xylobanus sp.	UPOL 000154	Laos	KC538164	KC537955	-	KC538281	-
Cautires sp.	UPOL 000164	Laos	KC538165	KC537956	KC538667	KC538282	KC538473
Cautires sp.	UPOL 000174	Malaysia	KC538166	KC537957	-	KC538283	KC538474
Cautires sp.	UPOL 000177	Malaysia	KC538167	-	KC538668	KC538284	KC538475
Cautires sp.	UPOL 000178	Malaysia	KC538168	KC537958	KC538669	KC538285	KC538476
Xylobanus sp.	UPOL 000184	Borneo	KC538169	KC537959	KC538670	KC538286	KC538477
Cautires sp.	UPOL 000188	Laos	KC538170	KC537960	KC538671	KC538287	KC538478
Cautires sp.	UPOL 000189	Laos	KC538171	KC537961	KC538672	KC538288	KC538479
Cautires sp.	UPOL 000195	South Africa	KC538172	KC537962	KC538673	KC538289	KC538480
Microtrichalus sp.	UPOL 000199	Sulawesi	KC538173	KC537963	KC538674	KC538290	KC538481
Cautires sp.	UPOL 000205	Sumatra	KC538174	KC537964	KC538675	KC538291	KC538482
Cautires sp.	UPOL 000206	Sumatra	KC538175	KC537965	KC538676	KC538292	KC538483
Leptotrichalus sp.	UPOL 000208	Borneo	DQ181064	DQ181138	KC538677	DQ181212	DQ181366
Cautires sp.	UPOL 000217	Japan	KC538176	KC537966	KC538678	KC538293	KC538484
Cautires sp.	UPOL 000219	Japan	KC538177	KC537967	KC538679	KC538294	KC538485
Cautires sp.	UPOL 000220	Japan	KC538178	KC537968	KC538680	KC538295	KC538486
Xylobanus sp.	UPOL 000221	Japan	KC538179	-	KC538681	KC538296	KC538487
Xylobanus sp.	UPOL 000224	Japan	KC538180	KC537969	KC538682	KC538297	KC538488
Cautires sp.	UPOL 000246	Sumatra	KC538181	KC537970	KC538683	KC538298	KC538489
Metanoeus sp.	UPOL 000248	Sumatra	KC538182	KC537971	KC538684	KC538299	KC538490
Xylobanus sp.	UPOL 000262	Borneo	-	KC537972	KC538685	KC538300	KC538491
Xylobanus sp.	UPOL 000274	Borneo	KC538183	KC537973	KC538686	KC538301	KC538492
Cautires sp.	UPOL 000290	Laos	KC538184	KC537974	KC538687	KC538302	KC538493
Cautires sp.	UPOL 000294	Sumatra	KC538185	KC537975	KC538688	KC538303	KC538494
Cautires sp.	UPOL 000295	Sumatra	KC538186	KC537976	KC538689	KC538304	KC538495
Cautires sp.	UPOL 000297	Sumatra	KC538187	KC537977	KC538690	KC538305	KC538496
Cautires sp.	UPOL 000314	Sumatra	KC538188	KC537978	KC538691	KC538306	KC538497
Xylobanus sp.	UPOL 000315	Sumatra	KC538189	KC537979	KC538692	KC538307	KC538498
Cautires sp.	UPOL 000335	Borneo	-	-	KC538693	KC538308	KC538499
Cautires sp.	UPOL 000339	Borneo	KC538190	KC537980	KC538694	KC538309	KC538500
Cautires sp.	UPOL 000342	Borneo	KC538191	KC537981	KC538695	KC538310	KC538501
Cautires sp.	UPOL 000346	Borneo	KC538192	KC537982	KC538696	KC538311	KC538502
Cautires sp.	UPOL 000355	Jawa	-	KC537983	KC538697	KC538312	KC538503
Metriorrh. palawensis	UPOL 000366	Palawan	-	-	KC538698	DQ144665	DQ144691
Porrostoma rhipidum	UPOL 000372	Australia	KC538193	KC537984	KC538699	DQ144678	DQ144702
Microtrichalus sp.	UPOL 000373	Australia	KC538194	KC537985	KC538700	KC538313	KC538504
Metriorrhynchus sp.	UPOL 000374	Australia	KC538195	KC537986	KC538701	KC538314	KC538505
Microtrichalus sp.	UPOL 000375	Australia	KC538196	KC537987	KC538702	KC538315	KC538506
Microtrichalus sp.	UPOL 000376	Australia	KC538197	KC537988	KC538703	KC538316	KC538507
Porrost. haemorrhoidalis	UPOL 000378	Australia	KC538198	KC537989	KC538704	DQ144679	DQ144703
Xylobanus sp.	UPOL 000379	Palawan	KC538199	-	KC538705	KC538317	KC538508
Cautiromimus sp.	UPOL 000388	Palawan	-	-	-	KC538318	KC538509

Cautires sp.	UPOL 000395	Palawan	-	-	KC538706	KC538319	KC538510
Leptotrichalus sp.	UPOL 000396	Palawan	-	-	KC538707	KC538320	KC538511
Xylobanus sp.	UPOL 000402	Palawan	KC538200	-	KC538708	KC538322	KC538513
Cautires sp.	UPOL 000403	Palawan	-	KC537990	KC538709	KC538323	KC538514
Cautires sp.	UPOL 000411	Palawan	KC538201	-	KC538710	KC538324	KC538515
Microtrichalus sp.	UPOL 000412	Palawan	KC538202	KC537991	KC538711	KC538325	KC538516
Leptotrichalus sp.	UPOL 000419	Palawan	KC538203	KC537992	KC538712	KC538326	KC538517
Cautires sp.	UPOL 000425	Palawan	-	-	KC538713	KC538327	KC538518
Metanoeus sp.	UPOL 000434	Palawan	-	-	KC538714	KC538328	KC538519
Cautires sp.	UPOL A00017	Taiwan	-	KC537824	-	-	-
Xylobanus sp.	UPOL A00018	Taiwan	KC538033	KC537825	HQ456946	HQ456964	HQ456988
Cautires sp.	UPOL A00019	Taiwan	KC538034	KC537826	KC538715	KC538329	KC538520
Cautires sp.	UPOL A00020	Taiwan	KC538035	KC537827	-	-	-
Cautires sp.	UPOL A00021	Taiwan	KC538036	KC537828	HQ456947	HQ456965	-
Cautires sp.	UPOL A00022	Madagascar	KC538037	KC537829	KC538716	HQ456966	HQ456986
Cautires sp.	UPOL A00023	Madagascar	KC538038	KC537830	KC538717	KC538330	KC538521
Cautires sp.	UPOL A00024	Madagascar	KC538039	KC537831	KC538718	KC538331	KC538522
Cautires sp.	UPOL A00025	Madagascar	KC538040	KC537832	KC538719	KC538332	KC538523
Cautires sp.	UPOL A00026	Madagascar	KC538041	KC537833	-	KC538333	KC538524
Cautires sp.	UPOL A00027	Madagascar	KC538042	KC537834	-	KC538334	KC538525
Cautires sp.	UPOL A00028	Madagascar	KC538043	KC537835	-	KC538335	-
Cautires sp.	UPOL A00029	Madagascar	KC538044	KC537836	-	KC538336	KC538526
Cautires sp.	UPOL A00030	Madagascar	KC538045	KC537837	KC538720	KC538337	KC538527
Synchonnus sp.	UPOL A00031	Australia	KC538046	KC537838	KC538721	KC538338	KC538528
Trichalus sp.	UPOL A00032	Australia	KC538047	KC537839	KC538722	KC538339	KC538529
Ditua sp.	UPOL A00033	Australia	KC538048	KC537840	KC538723	-	KC538530
Metriorrhynchus sp.	UPOL A00034	Australia	KC538049	KC537841	KC538724	KC538340	KC538531
Porrostoma sp.	UPOL A00035	Australia	KC538050	KC537842	KC538725	KC538341	KC538532
Porrostoma sp.	UPOL A00036	Australia	KC538051	KC537843	KC538726	KC538342	KC538533
Porrostoma sp.	UPOL A00037	Australia	KC538052	KC537844	KC538727	KC538343	KC538534
Metriorrhynchus sp.	UPOL A00038	Australia	KC538053	KC537845	KC538728	KC538344	KC538535
Metriorrhynchus sp.	UPOL A00039	Australia	KC538054	KC537846	KC538729	KC538345	KC538536
Porrostoma sp.	UPOL A00040	Australia	KC538055	KC537847	KC538730	KC538346	KC538537
Porrostoma sp.	UPOL A00041	Australia	KC538056	KC537848	KC538731	KC538347	KC538538
Porrostoma sp.	UPOL A00042	Australia	KC538057	KC537849	-	KC538348	KC538539
Metriorrhynchus sp.	UPOL A00043	Australia	KC538058	KC537850	KC538732	KC538349	KC538540
Porrostoma sp.	UPOL A00044	Australia	KC538059	KC537851	KC538733	KC538350	KC538541
Porrostoma sp.	UPOL A00045	Australia	KC538060	KC537852	KC538734	KC538351	KC538542
Metriorrhynchus sp.	UPOL A00046	Australia	KC538061	KC537853	KC538735	KC538352	KC538543
Cautires sp.	UPOL A00048	Malaysia	KC538063	KC537855	HQ456948	HQ456967	HQ456990
Metriorrhynchus sp.	UPOL A00049	Malaysia	KC538064	KC537856	KC538736	KC538354	KC538545
Cautires sp.	UPOL A00050	Malaysia	KC538065	KC537857	-	-	KC538546
Leptotrichalus sp.	UPOL A00052	Philippines	KC538066	KC537858	HQ456949	HQ456968	HQ456991
Xylobanus sp.	UPOL A00053	Palawan	KC538067	KC537859	KC538737	KC538355	KC538547
Xylobanus sp.	UPOL A00054	Palawan	KC538068	-	KC538738	-	KC538548
Cautires sp.	UPOL A00057	Philippines	KC538069	KC537860	-	KC538356	KC538549
Cautires sp.	UPOL A00058	Philippines	KC538070	KC537861	-	KC538357	KC538550
Leptotrichalus sp.	UPOL A00059	Philippines	KC538071	KC537862	KC538739	KC538358	KC538551
Leptotrichalus sp.	UPOL A00061	Philippines	KC538073	KC537864	KC538741	KC538360	KC538553
Cautires sp.	UPOL A00062	Philippines	KC538074	KC537865	KC538742	KC538361	KC538554
Metanoeus sp.	UPOL A00063	Philippines	KC538075	KC537866	-	KC538362	KC538555
Metanoeus sp.	UPOL A00064	Philippines	KC538076	KC537867	-	KC538363	KC538556
Metanoeus sp.	UPOL A00065	Philippines	KC538077	KC537868	-	KC538364	KC538557
Sulabanus sp.	UPOL A00066	Philippines	KC538078	KC537869	KC538743	KC538365	KC538558
Sulabanus sp.	UPOL A00067	Philippines	KC538079	KC537870	KC538744	KC538366	KC538559
Microtrichalus sp.	UPOL A00068	Philippines	KC538080	KC537871	KC538745	KC538367	KC538560
Microtrichalus sp.	UPOL A00069	Philippines	KC538081	KC537872	KC538746	KC538368	KC538561
Sulabanus sp.	UPOL A00070	Philippines	KC538082	KC537873	KC538747	KC538369	KC538562

Sulabanus sp.	UPOL A00071	Philippines	KC538083	KC537874	KC538748	KC538370	KC538563
Microtrichalus sp.	UPOL A00073	Philippines	KC538084	KC537875	KC538749	KC538371	-
Xylobanus sp.	UPOL A00074	Philippines	KC538085	KC537876	KC538750	-	-
Sulabanus sp.	UPOL A00075	Philippines	KC538086	KC537877	KC538751	KC538372	KC538564
Xylobanus sp.	UPOL 00A076	Philippines	KC538087	KC537878	KC538752	KC538373	KC538565
Sulabanus sp.	UPOL A00077	Philippines	KC538088	KC537879	KC538753	KC538374	KC538566
Cautires sp.	UPOL A00078	Cameroon	KC538089	KC537880	KC538754	KC538375	KC538567
Cautires sp.	UPOL A00079	Cameroon	KC538090	KC537881	KC538755	KC538376	KC538568
Cautires sp.	UPOL A00080	Cameroon	KC538091	KC537882	HQ456950	HQ456969	HQ456992
Cautires sp.	UPOL A00081	Cameroon	KC538093	KC537884	KC538756	KC538377	KC538569
Cautires sp.	UPOL A00082	Cameroon	KC538092	KC537883	KC538757	KC538378	-
Cautires sp.	UPOL A00083	Cameroon	KC538094	KC537885	KC538758	KC538379	KC538570
Cautires sp.	UPOL A00084	Cameroon	KC538095	KC537886	KC538759	KC538380	KC538571
Cautires sp.	UPOL 00A085	Cameroon	KC538096	KC537887	KC538760	KC538381	KC538572
Cautires sp.	UPOL A00086	Cameroon	KC538097	KC537888	KC538761	KC538382	KC538573
Cautires sp.	UPOL A00087	Cameroon	KC538098	KC537889	KC538762	KC538383	KC538574
Cautires sp.	UPOL A00088	Cameroon	KC538099	KC537890	KC538763	KC538384	KC538575
Cautires sp.	UPOL A00089	Cameroon	KC538100	KC537891	KC538764	KC538385	KC538576
Cautires sp.	UPOL A00090	Cameroon	KC538101	KC537892	KC538765	KC538386	KC538577
Xylobanus sp.	UPOL A00091	Cameroon	KC538102	KC537893	-	KC538387	KC538578
Cautires sp.	UPOL A00092	Cameroon	KC538103	KC537894	KC538766	KC538388	KC538579
Cautires sp.	UPOL A00093	Cameroon	KC538104	KC537895	KC538767	KC538389	KC538580
Xylobanus sp.	UPOL A00094	Cameroon	KC538105	KC537896	KC538768	KC538390	KC538581
Xylobanus sp.	UPOL A00095	Cameroon	KC538106	KC537897	KC538769	-	KC538582
Xylobanus sp.	UPOL A00096	Cameroon	KC538107	KC537898	KC538770	-	KC538583
Xylobanus sp.	UPOL A00097	Cameroon	KC538108	KC537899	KC538771	KC538391	KC538584
Xylobanus sp.	UPOL A00098	Cameroon	KC538109	KC537900	KC538772	KC538392	KC538585
Cautires sp.	UPOL A00099	Cameroon	KC538110	KC537901	KC538773	KC538393	KC538586
Cautires sp.	UPOL A00100	Cameroon	KC538111	-	KC538774	KC538394	KC538587
Cautires sp.	UPOL A00101	Cameroon	KC538112	KC537902	-	KC538395	-
Cautires sp.	UPOL A00102	Cameroon	KC538113	KC537903	-	KC538396	KC538588
Cautires sp.	UPOL A00103	Cameroon	KC538114	KC537904	-	KC538397	-
Xylobanus sp.	UPOL A00104	Cameroon	KC538115	KC537905	KC538775	-	KC538589
Cautires sp.	UPOL A00105	Cameroon	KC538116	KC537906	KC538776	-	KC538590
Cautires sp.	UPOL A00106	Cameroon	KC538117	KC537907	KC538777	KC538398	KC538591
Cautires sp.	UPOL A00107	Cameroon	KC538118	KC537908	-	KC538399	-
Cautires sp.	UPOL A00109	Cameroon	KC538119	KC537909	KC538778	KC538400	KC538592
Cautires sp.	UPOL A00110	Cameroon	KC538120	KC537910	KC538779	-	KC538593
Cautires sp.	UPOL A00111	Cameroon	KC538121	KC537911	KC538780	KC538401	KC538594
Cautires sp.	UPOL A00112	Cameroon	KC538122	KC537912	KC538781	KC538402	KC538595
Metriorrhynchus lineatus	UPOL 000L05	Malaysia	DQ181040	DQ181114	DQ180966	DQ181188	DQ181342
Cautires sp.	UPOL 000L06	Malaysia	DQ181041	DQ181115	DQ180967	DQ181189	DQ181343
Cautires sp.	UPOL 000L14	South Africa	DQ181045	DQ181119	DQ180971	DQ181193	DQ181347
Microtrichalus sp.	UPOL 000L23	Malaysia	DQ181052	DQ181126	DQ180978	DQ181200	DQ181354
Xylobanus kundratai	UPOL MD0029	Sulawesi	KC538204	KC537993	-	HQ456972	HQ456994
Sulabanus lalui	UPOL MD0030	Sulawesi	KC538205	KC537994	-	KC538403	KC538596
Sulabanus katarinae	UPOL MD0033	Sulawesi	KC538206	KC537995	KC538783	KC538404	KC538597
Sulabanus lineatus	UPOL MD0034	Sulawesi	KC538207	KC537996	KC538784	KC538405	KC538598
Xylobanus kundratai	UPOL MD0036	Sulawesi	KC538208	KC537997	-	HQ456973	HQ456995
Sulabanus mamasensis	UPOL MD0044	Sulawesi	KC538209	KC537998	KC538785	KC538406	KC538599
Sulabanus gracilis	UPOL MD0064	Sulawesi	KC538210	KC537999	KC538786	KC538407	KC538600
Sulabanus similis	UPOL MD0065	Sulawesi	KC538211	KC538000	-	KC538408	KC538601
Sulabanus gracilis	UPOL MD0067	Sulawesi	KC538212	KC538001	KC538787	KC538409	KC538602
Sulabanus cordatus	UPOL MD0069	Sulawesi	KC538213	KC538002	KC538788	KC538410	KC538603
Sulabanus gracilis	UPOL MD0071	Sulawesi	KC538214	KC538003	KC538789	KC538411	KC538604
Sulabanus cordatus	UPOL MD0081	Sulawesi	KC538215	KC538004	KC538790	KC538412	KC538605
Microtrichalus sp.	UPOL MD0097	Sulawesi	KC538216	KC538005	KC538791	HQ456978	HQ457000
Microtrichalus sp.	UPOL MD0098	Sulawesi	KC538217	KC538006	HQ456956	HQ456979	HQ457001

Broxylus pfeifferi	UPOL MD0099	Sulawesi	KC538218	KC538007	HQ456957	HQ456980	HQ457002
Broxylus malinensis	UPOL MD0101	Sulawesi	KC538219	KC538008	HQ456958	HQ456981	HQ457003
Broxylus pendolensis	UPOL MD0106	Sulawesi	KC538220	KC538009	KC538792	KC538413	KC538606
Broxylus kalamensis	UPOL MD0107	Sulawesi	KC538221	KC538010	KC538793	KC538414	KC538607
Wakarumbia pendolensis	UPOL MD0109	Sulawesi	KC538222	KC538011	KC538794	KC538415	KC538608
Wakarumbia monacha	UPOL MD0111	Sulawesi	KC538223	KC538012	KC538795	KC538416	KC538609
Wakarumbia petri	UPOL MD0118	Sulawesi	KC538224	KC538013	HQ456960	HQ456983	HQ457005
Wakarumbia montana	UPOL MD0119	Sulawesi	KC538225	KC538014	HQ456961	HQ456984	HQ457006
Wakarumbia fasciata	UPOL MD0121	Sulawesi	KC538226	KC538015	KC538796	KC538418	KC538610
Wakarumbia sp.	UPOL MD0126	Sulawesi	KC538227	KC538016	KC538797	KC538419	KC538611
Wakarumbia grisea	UPOL MD0127	Sulawesi	KC538228	KC538017	-	KC538420	KC538612
Wakarumbia nepeensis	UPOL MD0129	Sulawesi	KC538229	KC538018	KC538798	KC538421	KC538613
Wakarumbia kundratai	UPOL MD0130	Sulawesi	KC538230	KC538019	KC538799	KC538422	KC538614
Wakarumbia sp.	UPOL MD0132	Sulawesi	KC538231	KC538020	KC538800	KC538423	KC538615
Wakarumbia kalamensis	UPOL MD0133	Sulawesi	KC538232	KC538021	KC538801	KC538424	KC538616
Wakarumbia linearis	UPOL MD0134	Sulawesi	KC538233	KC538022	KC538802	KC538425	KC538617
Wakarumbia nepeensis	UPOL MD0135	Sulawesi	KC538234	KC538023	KC538803	KC538426	KC538618
Wakarumbia aurea	UPOL MD0136	Sulawesi	KC538235	KC538024	KC538804	KC538427	KC538619
Wakarumbia aurea	UPOL MD0137	Sulawesi	KC538236	KC538025	KC538805	KC538428	KC538620
Wakarumbia fascicularis	UPOL MD0140	Sulawesi	KC538237	KC538026	KC538806	KC538429	KC538621
Wakarumbia pendolensis	UPOL MD0143	Sulawesi	KC538238	KC538027	KC538807	KC538430	KC538622
Wakarumbia fascicularis	UPOL MD0145	Sulawesi	KC538239	KC538028	KC538808	KC538431	KC538623
Wakarumbia mamasensis	UPOL MD0155	Sulawesi	KC538240	KC538029	KC538809	KC538432	KC538624
Wakarumbia grisea	UPOL MD0156	Sulawesi	KC538241	KC538030	KC538810	KC538433	KC538625
Wakarumbia mamasensis	UPOL MD0157	Sulawesi	KC538242	KC538031	KC538811	KC538434	KC538626
Wakarumbia kalamensis	UPOL MD0169	Sulawesi	KC538243	KC538032	KC538812	KC538435	KC538627

Remark. DQ and HQ sequences were published in the previous studies on net-winged beetles (Bocak et al., 2008, Bocak & Yagi 2010; Kubecek et al. 2011). The KC sequences are published here for the first time.

The voucher specimens are deposited in the Laboratory of Molecular Systematics, Department of Zoology, Palacky University, Olomouc, Czech Republic.
