# Supplementary material for: Phylogeography of Poorly Dispersing Net-Winged Beetles: A Role of Drifting India in the Origin of Afrotropical and Oriental Fauna
Source: PLoS One. 2013 Jun 26;8(6):e67957. doi: 10.1371/journal.pone.0067957 (PMC3694047; doi:10.1371/journal.pone.0067957)
Supplement: Table S2 — (PDF) [file pone.0067957.s004.pdf]

Table S2. Primers and conditions used for PCR amplifications

Fragment	Code	-mer	Sequence (5’ >> 3’)
18S rRNA	5’	24	GACAACCTGGTTGATCCTGCCAGT
	b5.0	19	TAACCGCAACAACTTTAAT
	ai	22	CCTGAGAAACGGCTACCACATC
	b2.5	20	TCTTTGGCAAATGCTTTCGC
	a1.0	20	GGTGAAATTCTTGGACCGTC
	bi	20	GAGTCTCGTTCGTTATCGGA
	3’I	24	CACCTACGGAAACCTTGTTACGAC
	a2.0	19	ATGGTTGCAAAGCTGAAAC
28S rRNA	ff	20	TTACACACTCCTTAGCGGAT
	dd	19	GGGACCCGTCTTGAAACAC
16S rDNA	16a	20	CGCCTGTTTAACAAAAACAT
	16b	22	CCGGTCTGAACTCAGATCATGT
	ND1A	27	GGTCCCTTACGAATTTGAATATATCCT
cox1 mtDNA	JerM	23	CAACAYYTATTTTGRTTYTTTGG
	Pat	25	TCCATTGCACTAATCTGCCATATTA
	Marilyn	21	TCATAAGTTCAGTATCATTG
	Marcy	27	TARTTCRTATGWTCAATAYCAYTGRTG
nad5 mtDNA	OF1	29	CCTACTCCTGTTTCTGCTTTAGTTCATTC
	R6	29	GAAACGAAAAATCGTATTTAATTTCGACT
