# Supplementary material for: Phylogeography of Poorly Dispersing Net-Winged Beetles: A Role of Drifting India in the Origin of Afrotropical and Oriental Fauna
Source: PLoS One. 2013 Jun 26;8(6):e67957. doi: 10.1371/journal.pone.0067957 (PMC3694047; doi:10.1371/journal.pone.0067957)
Supplement: Table S3 — (PDF) [file pone.0067957.s005.pdf]

Table S3. The length of DNA fragments and the numbers of informative characters in datasets (gaps as 5th character).

Datasets		All data	18S rDNA	28S rDNA	*rrnl-tRNA-nad1*	*coi-tRNAcoii*	*nad5-tRNAs*
# of specimens		249	244	240	227	241	242
Clustal W	# of characters	5792	1913	655	840	1100	1284
	parsimony informative	2233	212	95	416	621	889
BlastAlign	# of characters	5963	1947	668	947	1101	1300
	parsimony informative	2163	205	92	397	621	848
Muscle	# of characters	5827	1921	657	858	1099	1292
	parsimony informative	2220	207	95	411	622	885
T-Coffee	# of characters	5881	1936	669	866	1101	1309
	parsimony informative	2216	208	92	413	621	882
Mafft	# of characters	5822	1909	659	858	1099	1297
	parsimony informative	2243	221	96	418	622	886
