# Supplementary material for: Phylogeography of Poorly Dispersing Net-Winged Beetles: A Role of Drifting India in the Origin of Afrotropical and Oriental Fauna
Source: PLoS One. 2013 Jun 26;8(6):e67957. doi: 10.1371/journal.pone.0067957 (PMC3694047; doi:10.1371/journal.pone.0067957)
Supplement: Table S4 — (PDF) [file pone.0067957.s006.pdf]

Table S4. Results of the BayesTrait analysis (all analyzed clades)

Node	Defined range:								
#	Sundas	Sulawesi	Contin. Asia	outside	Africa	Australia	Madagascar	Philippines	Palawan
18	0,32807	0,02622	0,08885	0	0,00079	0,02615	0,02385	0,32955	0,17649
20	0,48098	0,00626	0,11454	0	0,00006	0,00757	0,03428	0,21455	0,14177
55	0	0,00387	0	0	0,98903	0	0,00481	0,00011	0,00209
63	0,00022	0,00381	0,00284	0	0	0,02506	0,96792	0	0
64	0,08706	0,02045	0,06322	0	0,00219	0,00962	0,66407	0,09364	0,05975
67	0,21414	0,00106	0,00903	0	0	0,00164	0,01456	0,09429	0,66526
68	0,51419	0,00202	0,02937	0	0	0,00268	0,00925	0,10771	0,33477
69	0,77092	0,00086	0,02434	0	0	0,00188	0,00984	0,07595	0,11620
70	0,22706	0,00199	0,55502	0	0,00011	0,00124	0,03854	0,07972	0,09630
76	0,09651	0,00057	0,83519	0	0	0,00009	0,00489	0,02192	0,04084
77	0,19072	0,00199	0,63056	0	0,00040	0,00222	0,04846	0,05009	0,07553
78	0,13407	0	0,78690	0	0,00017	0,00047	0,01920	0,01637	0,04276
79	0,20077	0,00190	0,71169	0	0	0,00264	0,02640	0,03205	0,02453
80	0,17154	0,00314	0,64603	0	0,00015	0,00685	0,05849	0,05146	0,06231
81	0,12858	0,00226	0,67799	0	0	0,00091	0,03355	0,08261	0,07406
82	0,17365	0,00181	0,64440	0	0,00057	0,00189	0,02065	0,08317	0,07384
83	0,07133	0,00078	0,79451	0	0,00021	0,00236	0,05306	0,03991	0,03780
84	0,10204	0,00164	0,77232	0	0	0,01275	0,02762	0,03595	0,04760
86	0,28323	0,00086	0,53106	0	0	0,00166	0,05054	0,06350	0,06909
87	0,09733	0,00013	0,81795	0	0,00025	0,00077	0,02967	0,01744	0,03644
90	0,24255	0,00143	0,55269	0	0	0,00168	0,03407	0,06318	0,10433
91	0,40033	0,00117	0,40022	0	0,00029	0,00092	0,03768	0,06553	0,09384
93	0,64606	0,00456	0,23739	0	0	0,00048	0,01242	0,05356	0,04553
94	0,30633	0,00142	0,50025	0	0,00598	0,00226	0,03225	0,07021	0,08125
103	0,68382	0,00214	0,04240	0	0	0,00723	0,01594	0,09352	0,15494
105	0,22618	0,00243	0,02942	0	0	0,01007	0,00692	0,20295	0,52185
106	0,08102	0,00708	0,01548	0	0	0,00659	0,03948	0,40616	0,44410

110	0,26134	0,00250	0,01838	0	0,00010	0,00250	0,03788	0,33747	0,33982
111	0,53239	0,00112	0,02711	0	0	0,00077	0,01590	0,16576	0,25693
112	0,36663	0,00086	0,34155	0	0	0,00160	0,01535	0,12090	0,15309
118	0,46716	0,00220	0,21820	0	0,00008	0,00280	0,03732	0,12201	0,15021
119	0,62822	0,00404	0,16709	0	0	0,00036	0,01782	0,09419	0,08827
120	0,52113	0,00013	0,37383	0	0	0,00059	0,01297	0,03624	0,05510
121	0,34379	0,00718	0,45107	0,00007	0	0,00313	0,03360	0,07088	0,09028
122	0,20496	0,00075	0,70341	0	0,00020	0,00212	0,01728	0,03938	0,03191
123	0,22740	0,00064	0,62138	0,00001	0,00001	0,00533	0,01681	0,05439	0,07403
124	0,16606	0,00004	0,76362	0	0	0,00006	0,01782	0,01323	0,03917
125	0,07343	0,00026	0,82507	0,00001	0	0,00030	0,02258	0,03902	0,03932
127	0,05744	0,00032	0,37550	0	0,00303	0,00024	0,36568	0,08801	0,10978
130	0,12499	0,00867	0,39791	0,00010	0,00114	0,03012	0,12532	0,12240	0,18934
131	0,11955	0,03122	0,25048	0,00001	0,00049	0,03260	0,06076	0,24317	0,26172
132	0,00044	0,00008	0,00012	0	0	0,00098	0,00131	0,24421	0,75287
134	0,05943	0,01111	0,66261	0,00002	0,00007	0,01264	0,05847	0,08921	0,10644
135	0,14776	0,00407	0,64523	0,00004	0,00011	0,00348	0,04870	0,06977	0,08085
137	0,03404	0,28229	0,32691	0,00016	0,00003	0,10239	0,06841	0,05246	0,13332
139	0,06002	0,01562	0,69908	0,00002	0,00001	0,02758	0,01034	0,06700	0,12033
140	0,05814	0,01505	0,61849	0	0	0,01863	0,04806	0,09906	0,14257
141	0,02893	0,06566	0,50941	0	0,00004	0,00053	0,20388	0,08926	0,10228
142	0,07070	0,00157	0,36993	0	0,01400	0,00132	0,15853	0,17732	0,20662
143	0,00840	0,00219	0,02768	0,00037	0,00022	0,84651	0,01111	0,04060	0,06292
146	0,00129	0,86213	0,00217	0,00008	0,00166	0,06784	0,00586	0,03172	0,02726
153	0,03697	0,59083	0,04589	0,00001	0,00016	0,04965	0,01823	0,13174	0,12652
154	0,00702	0,83416	0,03789	0	0,00027	0,01710	0,00324	0,04239	0,05794
155	0,01464	0,54652	0,00389	0	0	0,04770	0,01044	0,10646	0,27035
160	0,00037	0,00704	0,00047	0,00002	0	0,94944	0,00034	0,02348	0,01883
172	0,00358	0,74455	0,00472	0,00001	0,00063	0,06641	0,00850	0,08855	0,08304
178	0	0,99159	0	0	0	0,00378	0,00023	0,00063	0,00371
179	0,00055	0,89868	0,00242	0	0,00001	0,02639	0,01446	0,03295	0,02454
183	0,00011	0,89517	0,00042	0	0,00001	0,01407	0,00314	0,05800	0,02909
184	0	0,03997	0,00012	0	0	0,93317	0,01446	0,00139	0,01085
206	0	0,99198	0	0	0	0,00178	0,00379	0,001676	0,00072

207	0,01163	0,00291	0,01103	0	0,00003	0,00536	0,01503	0,44108	0,51294
208	0,17242	0,00227	0,02063	0	0,00007	0,00379	0,01377	0,37242	0,41463
209	0,02165	0,01357	0,01630	0,00002	0,00005	0,01228	0,02532	0,42832	0,48250
210	0,01358	0,00608	0,01447	0	0,00006	0,00686	0,00919	0,70429	0,24546
211	0,01821	0,00179	0,04295	0	0,00001	0,00161	0,01503	0,48930	0,43111
212	0,00587	0,53613	0,00795	0,00001	0,00117	0,08395	0,01566	0,20606	0,14320
213	0,00099	0,05712	0,00170	0,00042	0,00019	0,84897	0,01586	0,04423	0,03052
217	0,00063	0,00015	0,00002	0	0	0,00013	0,00321	0,49732	0,49853
218	0,23456	0,00065	0,00454	0	0	0,00046	0,00270	0,37956	0,37752
219	0,01306	0,00012	0,00062	0	0	0,00004	0,00380	0,82981	0,15255
220	0,00020	0,55103	0,00288	0	0,00011	0,00818	0,01045	0,27679	0,15036
223	0	0,00289	0,00019	0	0	0,96642	0,00388	0,01271	0,01386
224	0,00018	0,00168	0,00190	0,00029	0	0,97036	0,00238	0,01971	0,00349
225	0,00008	0,06730	0,00025	0,00001	0,00001	0,90288	0,01954	0,00823	0,00170
226	0,00053	0,00029	0	0	0,00013	0,99138	0,00074	0,00629	0,00064
227	0,00004	0,00003	0,00002	0,00002	0,00017	0,94138	0,00008	0,01453	0,04373
228	0,30559	0,01200	0,06945	0,00002	0,00085	0,00907	0,04290	0,33928	0,22083
230	0,26207	0,00924	0,44753	0,00003	0,00030	0,01237	0,05743	0,09634	0,11468
234	0,22765	0,00795	0,02062	0	0,00001	0,00413	0,01604	0,52249	0,20110
235	0,46743	0,00369	0,04002	0	0,00001	0,00384	0,01294	0,32298	0,14908
236	0,72397	0,00093	0,03026	0	0	0,00054	0,03204	0,12209	0,09017
237	0,76160	0,00073	0,03055	0	0,00036	0,00019	0,00868	0,10000	0,09790
238	0,49536	0,00256	0,03253	0	0,00002	0,00480	0,01057	0,12257	0,33158
239	0,25720	0,03002	0,33029	0,00026	0,00204	0,03302	0,07744	0,11255	0,15719
240	0,00615	0,00216	0,10669	0	0	0,75762	0,02838	0,03024	0,06877
241	0,05665	0,01317	0,15450	0,00007	0,00016	0,57861	0,01155	0,14325	0,04203
425	0,04622	0,00002	0,54908	0	0,05274	0,00086	0,24338	0,03006	0,07766
426	0	0,00023	0	0	0	0,92463	0,00094	0,07371	0,00039

NODE18 000069, A00058, A00057
NODE20 000069, A00058, A00057, 000205, 000335
NODE55 A00090, A00080, A00099, A00105, A00082, A00101, A00103, A00081, A00107, 000195, 000L14, A00078, A00085, A00100, A00111, A00112, A00110, A00109, A00088, A00087, A00106, A00102, A00093, A00089, A00079, A00084, A00083, A00086, A00092, A00094, A00097, A00098, A00091, A00096, A00104, A00095
NODE63 A00023, A00022, A00030, A00026, A00027, A00025, A00029, A00028, A00024
NODE64 000132, A00023, A00022, A00030, A00026, A00027, A00025, A00029, A00028, A00024
NODE67 000043, 000403
NODE68 000052, 000056, 000043, 000403
NODE69 000L06, 000052, 000056, 000043, 000403
NODE70 000066, 000L06, 000052, 000056, 000043, 000403
NODE76 000109, 000047, 000178, 000123, A00021, 000219, 000217
NODE77 000122, 000290
NODE78 000109, 000047, 000178, 000123, A00021, 000219, 000217, 000122, 000290
NODE79 000066, 000L06, 000052, 000056, 000043, 000403, 000109, 000047, 000178, 000123, A00021, 000219, 000217, 000122, 000290
NODE80 000068, 000030, 000066, 000L06, 000052, 000056, 000043, 000403, 000109, 000047, 000178, 000123, A00021, 000219, 000217, 000122, 000290
NODE81 000220, 000068, 000030, 000066, 000L06, 000052, 000056, 000043, 000403, 000109, 000047, 000178, 000123, A00021, 000219, 000217, 000122, 000290
NODE82 000147, 000174
NODE83 000189, 000147, 000174
NODE84 000188, 000189, 000147, 000174
NODE86 000075, A00050, A00048
NODE87 000064, 000075, A00050, A00048
NODE90 000044, 000104, A00017, 000164
NODE91 000044, 000104, A00017, 000164, 000048
NODE93 000044, 000104, A00017, 000164, 000048, 000050, 000060
NODE94 000064, 000075, A00050, A00048, 000044, 000104, A00017, 000164, 000048, 000050, 000060
NODE103 000411, 000355, 000262, 000297, 000294, 000342, 000339
NODE105 000074, 000425, 000395
NODE106 000074, 000425, 000395, A00062
NODE110 000074, 000425, 000395, A00062, 000346, 000088, 000315, 000206
NODE111 000411, 000355, 000262, 000297, 000294, 000342, 000339, 000074, 000425, 000395, A00062, 000346, 000088, 000315, 000206
NODE112 A00019, 000411, 000355, 000262, 000297, 000294, 000342, 000339, 000074, 000425, 000395, A00062, 000346, 000088, 000315, 000206

NODE118 A00019, 000411, 000355, 000262, 000297, 000294, 000342, 000339, 000074, 000425, 000395, A00062, 000346, 000088, 000315, 000206, 000040, 000037, 000177, 000314, 000295, 000246
NODE119 000084, 000080, 000081, 000090, A00019, 000411, 000355, 000262, 000297, 000294, 000342, 000339, 000074, 000425, 000395, A00062, 000346, 000088, 000315, 000206, 000040, 000037, 000177, 000314, 000295, 000246
NODE120 000064, 000075, A00050, A00048, 000044, 000104, A00017, 000164, 000048, 000050, 000060, 000084, 000080, 000081, 000090, A00019, 000411, 000355, 000262, 000297, 000294, 000342, 000339, 000074, 000425, 000395, A00062, 000346, 000088, 000315, 000206, 000040, 000037, 000177, 000314, 000295, 000246
NODE121 000079, 000064, 000075, A00050, A00048, 000044, 000104, A00017, 000164, 000048, 000050, 000060, 000084, 000080, 000081, 000090, A00019, 000411, 000355, 000262, 000297, 000294, 000342, 000339, 000074, 000425, 000395, A00062, 000346, 000088, 000315, 000206, 000040, 000037, 000177, 000314, 000295, 000246
NODE122 000188, 000189, 000147, 000174, 000079, 000064, 000075, A00050, A00048, 000044, 000104, A00017, 000164, 000048, 000050, 000060, 000084, 000080, 000081, 000090, A00019, 000411, 000355, 000262, 000297, 000294, 000342, 000339, 000074, 000425, 000395, A00062, 000346, 000088, 000315, 000206, 000040, 000037, 000177, 000314, 000295, 000246
NODE123 000188, 000189, 000147, 000174, 000079, 000064, 000075, A00050, A00048, 000044, 000104, A00017, 000164, 000048, 000050, 000060, 000084, 000080, 000081, 000090, A00019, 000411, 000355, 000262, 000297, 000294, 000342, 000339, 000074, 000425, 000395, A00062, 000346, 000088, 000315, 000206, 000040, 000037, 000177, 000314, 000295, 000246, 000070
NODE124 000220, 000068, 000030, 000066, 000L06, 000052, 000056, 000043, 000403, 000109, 000047, 000178, 000123, A00021, 000219, 000217, 000122, 000290, 000188, 000189, 000147, 000174, 000079, 000064, 000075, A00050, A00048, 000044, 000104, A00017, 000164, 000048, 000050, 000060, 000084, 000080, 000081, 000090, A00019, 000411, 000355, 000262, 000297, 000294, 000342, 000339, 000074, 000425, 000395, A00062, 000346, 000088, 000315, 000206, 000040, 000037, 000177, 000314, 000295, 000246, 000070
NODE125 000220, 000068, 000030, 000066, 000L06, 000052, 000056, 000043, 000403, 000109, 000047, 000178, 000123, A00021, 000219, 000217, 000122, 000290, 000188, 000189, 000147, 000174, 000079, 000064, 000075, A00050, A00048, 000044, 000104, A00017, 000164, 000048, 000050, 000060, 000084, 000080, 000081, 000090, A00019, 000411, 000355, 000262, 000297, 000294, 000342, 000339, 000074, 000425, 000395, A00062, 000346, 000088, 000315, 000206, 000040, 000037, 000177, 000314, 000295, 000246, 000070, A00020
NODE127 A00090, A00080, A00099, A00105, A00082, A00101, A00103, A00081, A00107, 000195, 000L14, A00078, A00085, A00100, A00111, A00112, A00110, A00109, A00088, A00087, A00106, A00102, A00093, A00089, A00079, A00084, A00083, A00086, A00092, A00094, A00097, A00098, A00091, A00096, A00104, A00095, 000132, A00023, A00022, A00030, A00026, A00027, A00025, A00029, A00028, A00024, 000220, 000068, 000030, 000066, 000L06, 000052, 000056, 000043, 000403, 000109, 000047, 000178, 000123, A00021, 000219, 000217, 000122, 000290, 000188, 000189, 000147, 000174, 000079, 000064, 000075, A00050, A00048, 000044, 000104, A00017, 000164, 000048, 000050, 000060, 000084, 000080, 000081, 000090, A00019, 000411, 000355, 000262, 000297, 000294, 000342, 000339, 000074, 000425, 000395, A00062, 000346, 000088, 000315, 000206, 000040, 000037, 000177, 000314, 000295, 000246, 000070, A00020
NODE130 000152, 000120, 000379
NODE131 A00074, A00053, 000152, 000120, 000379
NODE132 000402, A00054
NODE134 000402, A00054, 000154, A00018

NODE135 000274, 000224
NODE137 000153, MD0029, MD0036
NODE138 000274, 000224, 000153, MD0029, MD0036
NODE139 000402, A00054, 000154, A00018, 000274, 000224, 000153, MD0029, MD0036
NODE140 A00074, A00053, 000152, 000120, 000379, 000402, A00054, 000154, A00018, 000274, 000224, 000153, MD0029, MD0036
NODE141 A00090, A00080, A00099, A00105, A00082, A00101, A00103, A00081, A00107, 000195, 000L14, A00078, A00085, A00100, A00111, A00112, A00110, A00109, A00088, A00087, A00106, A00102, A00093, A00089, A00079, A00084, A00083, A00086, A00092, A00094, A00097, A00098, A00091, A00096, A00104, A00095, 000132, A00023, A00022, A00030, A00026, A00027, A00025, A00029, A00028, A00024, 000220, 000068, 000030, 000066, 000L06, 000052, 000056, 000043, 000403, 000109, 000047, 000178, 000123, A00021, 000219, 000217, 000122, 000290, 000188, 000189, 000147, 000174, 000079, 000064, 000075, A00050, A00048, 000044, 000104, A00017, 000164, 000048, 000050, 000060, 000084, 000080, 000081, 000090, A00019, 000411, 000355, 000262, 000297, 000294, 000342, 000339, 000074, 000425, 000395, A00062, 000346, 000088, 000315, 000206, 000040, 000037, 000177, 000314, 000295, 000246, 000070, A00020, A00074, A00053, 000152, 000120, 000379, 000402, A00054, 000154, A00018, 000274, 000224, 000153, MD0029, MD0036
NODE142 000069, A00058, A00057, 000205, 000335, A00090, A00080, A00099, A00105, A00082, A00101, A00103, A00081, A00107, 000195, 000L14, A00078, A00085, A00100, A00111, A00112, A00110, A00109, A00088, A00087, A00106, A00102, A00093, A00089, A00079, A00084, A00083, A00086, A00092, A00094, A00097, A00098, A00091, A00096, A00104, A00095, 000132, A00023, A00022, A00030, A00026, A00027, A00025, A00029, A00028, A00024, 000220, 000068, 000030, 000066, 000L06, 000052, 000056, 000043, 000403, 000109, 000047, 000178, 000123, A00021, 000219, 000217, 000122, 000290, 000188, 000189, 000147, 000174, 000079, 000064, 000075, A00050, A00048, 000044, 000104, A00017, 000164, 000048, 000050, 000060, 000084, 000080, 000081, 000090, A00019, 000411, 000355, 000262, 000297, 000294, 000342, 000339, 000074, 000425, 000395, A00062, 000346, 000088, 000315, 000206, 000040, 000037, 000177, 000314, 000295, 000246, 000070, A00020, A00074, A00053, 000152, 000120, 000379, 000402, A00054, 000154, A00018, 000274, 000224, 000153, MD0029, MD0036
NODE143 A00046, 000388
NODE146 MD0099, MD0107, MD0106, MD0101
NODE153 000011, 000017, A00049, 000009, 000L05
NODE154 000011, 000017, A00049, 000009, 000L05, 000010
NODE155 000366, 000011, 000017, A00049, 000009, 000L05, 000010
NODE160 000366, 000011, 000017, A00049, 000009, 000L05, 000010, 000374, A00043, A00034, A00036, A00037
NODE162 A00044, A00035, 000372
NODE163 A00040, A00044, A00035, 000372
NODE167 A00040, A00044, A00035, 000372, A00041, A00042, A00045, 000378
NODE168 000366, 000011, 000017, A00049, 000009, 000L05, 000010, 000374, A00043, A00034, A00036, A00037, A00040, A00044, A00035, 000372, A00041, A00042, A00045, 000378
NODE172 A00067, A00066, A00077, A00075, MD0065
NODE178 MD0081, MD0069, MD0034, MD0044, MD0064, MD0071, MD0067
NODE179 A00067, A00066, A00077, A00075, MD0065, MD0081, MD0069, MD0034, MD0044, MD0064, MD0071, MD0067

NODE182 MD0033, MD0030, A00071, A00070
NODE183 A00067, A00066, A00077, A00075, MD0065, MD0081, MD0069, MD0034, MD0044, MD0064, MD0071, MD0067, MD0033, MD0030, A00071, A00070
NODE184 000366, 000011, 000017, A00049, 000009, 000L05, 000010, 000374, A00043, A00034, A00036, A00037, A00040, A00044, A00035, 000372, A00041, A00042, A00045, 000378, A00067, A00066, A00077, A00075, MD0065, MD0081, MD0069, MD0034, MD0044, MD0064, MD0071, MD0067, MD0033, MD0030, A00071, A00070
NODE206 MD0127, MD0156, MD0111, MD0126, MD0136, MD0137, MD0109, MD0143, MD0121, MD0145, MD0140, MD0132, MD0135, MD0129, MD0134, MD0130, MD0119, MD0118, MD0169, MD0133, MD0157, MD0155
NODE207 000396, A00052
NODE208 000208, 000396, A00052
NODE209 000419, A00059
NODE210 000419, A00059, A00061
NODE211 000208, 000396, A00052, 000419, A00059, A00061
NODE212 MD0127, MD0156, MD0111, MD0126, MD0136, MD0137, MD0109, MD0143, MD0121, MD0145, MD0140, MD0132, MD0135, MD0129, MD0134, MD0130, MD0119, MD0118, MD0169, MD0133, MD0157, MD0155, 000208, 000396, A00052, 000419, A00059, A00061
NODE213 A00031, A00039, MD0127, MD0156, MD0111, MD0126, MD0136, MD0137, MD0109, MD0143, MD0121, MD0145, MD0140, MD0132, MD0135, MD0129, MD0134, MD0130, MD0119, MD0118, MD0169, MD0133, MD0157, MD0155, 000208, 000396, A00052, 000419, A00059, A00061
NODE217 000412, A00073
NODE218 000412, A00073, 000L23
NODE219 A00069, A00068, 000412, A00073, 000L23Microtrichalus
NODE220 MD0097, 000199, MD0098, A00069, A00068, 000412, A00073, 000L23
NODE223 MD0097, 000199, MD0098, A00069, A00068, 000412, A00073, 000L23, 000375, 000376, 000373
NODE224 A00032, MD0097, 000199, MD0098, A00069, A00068, 000412, A00073, 000L23, 000375, 000376, 000373
NODE225 A00031, A00039, MD0127, MD0156, MD0111, MD0126, MD0136, MD0137, MD0109, MD0143, MD0121, MD0145, MD0140, MD0132, MD0135, MD0129, MD0134, MD0130, MD0119, MD0118, MD0169, MD0133, MD0157, MD0155, 000208, 000396, A00052, 000419, A00059, A00061, A00032, MD0097, 000199, MD0098, A00069, A00068, 000412, A00073, 000L23, 000375, 000376, 000373
NODE226 000366, 000011, 000017, A00049, 000009, 000L05, 000010, 000374, A00043, A00034, A00036, A00037, A00040, A00044, A00035, 000372, A00041, A00042, A00045, 000378, A00067, A00066, A00077, A00075, MD0065, MD0081, MD0069, MD0034, MD0044, MD0064, MD0071, MD0067, MD0033, MD0030, A00071, A00070, A00031, A00039, MD0127, MD0156, MD0111, MD0126, MD0136, MD0137, MD0109, MD0143, MD0121, MD0145, MD0140, MD0132, MD0135, MD0129, MD0134, MD0130, MD0119, MD0118, MD0169, MD0133, MD0157, MD0155, 000208, 000396, A00052, 000419, A00059, A00061, A00032, MD0097, 000199, MD0098, A00069, A00068, 000412, A00073, 000L23, 000375, 000376, 000373
NODE227 A00046, 000388, A00038, A00033, MD0099, MD0107, MD0106, MD0101, 000366, 000011, 000017, A00049, 000009, 000L05, 000010, 000374, A00043, A00034, A00036, A00037, A00040, A00044, A00035, 000372, A00041, A00042, A00045, 000378, A00067, A00066, A00077, A00075, MD0065, MD0081, MD0069, MD0034, MD0044, MD0064, MD0071, MD0067, MD0033, MD0030, A00071, A00070, A00031, A00039, MD0127, MD0156, MD0111, MD0126, MD0136, MD0137, MD0109, MD0143, MD0121, MD0145, MD0140, MD0132, MD0135, MD0129, MD0134, MD0130,

MD0119, MD0118, MD0169, MD0133, MD0157, MD0155, 000208, 000396, A00052, 000419, A00059, A00061, A00032, MD0097, 000199, MD0098, A00069, A00068, 000412, A00073, 000L23, 000375, 000376, 000373
NODE228 A00076, 000071
NODE230 000221, A00076, 000071, 000184
NODE234 000026, A00065, A00063, A00064
NODE235 000121, 000026, A00065, A00063, A00064
NODE238 000125, 000105, 000248, 000121, 000026, A00065, A00063, A00064, 000434
NODE239 000221, A00076, 000071, 000184, 000125, 000105, 000248, 000121, 000026, A00065, A00063, A00064, 000434
NODE240 A00046, 000388, A00038, A00033, MD0099, MD0107, MD0106, MD0101, 000366, 000011, 000017, A00049, 000009, 000L05, 000010, 000374, A00043, A00034, A00036, A00037, A00040, A00044, A00035, 000372, A00041, A00042, A00045, 000378, A00067, A00066, A00077, A00075, MD0065, MD0081, MD0069, MD0034, MD0044, MD0064, MD0071, MD0067, MD0033, MD0030, A00071, A00070, A00031, A00039, MD0127, MD0156, MD0111, MD0126, MD0136, MD0137, MD0109, MD0143, MD0121, MD0145, MD0140, MD0132, MD0135, MD0129, MD0134, MD0130, MD0119, MD0118, MD0169, MD0133, MD0157, MD0155, 000208, 000396, A00052, 000419, A00059, A00061, A00032, MD0097, 000199, MD0098, A00069, A00068, 000412, A00073, 000L23, 000375, 000376, 000373, 000221, A00076, 000071, 000184, 000125, 000105, 000248, 000121, 000026, A00065, A00063, A00064, 000434
NODE241 000069, A00058, A00057, 000205, 000335, A00090, A00080, A00099, A00105, A00082, A00101, A00103, A00081, A00107, 000195, 000L14, A00078, A00085, A00100, A00111, A00112, A00110, A00109, A00088, A00087, A00106, A00102, A00093, A00089, A00079, A00084, A00083, A00086, A00092, A00094, A00097, A00098, A00091, A00096, A00104, A00095, 000132, A00023, A00022, A00030, A00026, A00027, A00025, A00029, A00028, A00024, 000220, 000068, 000030, 000066, 000L06, 000052, 000056, 000043, 000403, 000109, 000047, 000178, 000123, A00021, 000219, 000217, 000122, 000290, 000188, 000189, 000147, 000174, 000079, 000064, 000075, A00050, A00048, 000044, 000104, A00017, 000164, 000048, 000050, 000060, 000084, 000080, 000081, 000090, A00019, 000411, 000355, 000262, 000297, 000294, 000342, 000339, 000074, 000425, 000395, A00062, 000346, 000088, 000315, 000206, 000040, 000037, 000177, 000314, 000295, 000246, 000070, A00020, A00074, A00053, 000152, 000120, 000379, 000402, A00054, 000154, A00018, 000274, 000224, 000153, MD0029, MD0036, A00046, 000388, A00038, A00033, MD0099, MD0107, MD0106, MD0101, 000366, 000011, 000017, A00049, 000009, 000L05, 000010, 000374, A00043, A00034, A00036, A00037, A00040, A00044, A00035, 000372, A00041, A00042, A00045, 000378, A00067, A00066, A00077, A00075, MD0065, MD0081, MD0069, MD0034, MD0044, MD0064, MD0071, MD0067, MD0033, MD0030, A00071, A00070, A00031, A00039, MD0127, MD0156, MD0111, MD0126, MD0136, MD0137, MD0109, MD0143, MD0121, MD0145, MD0140, MD0132, MD0135, MD0129, MD0134, MD0130, MD0119, MD0118, MD0169, MD0133, MD0157, MD0155, 000208, 000396, A00052, 000419, A00059, A00061, A00032, MD0097, 000199, MD0098, A00069, A00068, 000412, A00073, 000L23, 000375, 000376, 000373, 000221, A00076, 000071, 000184, 000125, 000105, 000248, 000121, 000026, A00065, A00063, A00064, 000434
